# Whole Genome Analysis of Cyclin Dependent Kinase (*CDK*) Gene Family in Cotton and Functional Evaluation of the Role of *CDKF4* Gene in Drought and Salt Stress Tolerance in Plants

**DOI:** 10.3390/ijms19092625

**Published:** 2018-09-05

**Authors:** Richard Odongo Magwanga, Pu Lu, Joy Nyangasi Kirungu, Xiaoyan Cai, Zhongli Zhou, Xingxing Wang, Latyr Diouf, Yanchao Xu, Yuqing Hou, Yangguang Hu, Qi Dong, Kunbo Wang, Fang Liu

**Affiliations:** 1 Research Base in Anyang Institute of Technology, State Key Laboratory of Cotton Biology/Institute of Cotton Research, Chinese Academy of Agricultural Science (ICR, CAAS), Anyang 455000, China; magwangarichard@yahoo.com (R.O.M.); lupu1992@163.com (P.L.); joynk@cricaas.com.cn (J.N.K.); caixy@cricaas.com.cn (X.C.); zhouzl@cricaas.com.cn (Z.Z.); wangxx@cricaas.com.cn (X.W.); latyr@cricaas.com.cn (L.D.); xuyanchao2016@163.com (Y.X.); houyq@cricaas.com.cn (Y.H.); huygsh@163.com (Y.H.); dongqi@cricaas.com.cn (Q.D.); 2 School of Biological and Physical sciences (SBPS), Main campus, Jaramogi Oginga Odinga University of Science and Technology (JOOUST), P.O Box 210-40601, Bondo, Kenya

**Keywords:** cotton cyclin dependent kinase, segmental duplication, abiotic stress, plant growth and development, gene expression, transgenic arabidopsis lines, oxidant and antioxidant enzymes

## Abstract

Cotton (*Gossypium* spp.) is the number one crop cultivated for fiber production and the cornerstone of the textile industry. Drought and salt stress are the major abiotic stresses, which can have a huge economic impact on cotton production; this has been aggravated with continued climate change, and compounded by pollution. Various survival strategies evolved by plants include the induction of various stress responsive genes, such as cyclin dependent kinases (CDKs). In this study, we performed a whole-genome identification and analysis of the *CDK* gene family in cotton. We identified 31, 12, and 15 *CDK* genes in *G. hirsutum*, *G. arboreum*, and *G. raimondii* respectively, and they were classified into 6 groups. *CDK* genes were distributed in 15, 10, and 9 linkage groups of AD, D, and A genomes, respectively. Evolutionary analysis revealed that segmental types of gene duplication were the primary force underlying *CDK* genes expansion. RNA sequence and RT-qPCR validation revealed that *Gh_D12G2017 (CDKF4)* was strongly induced by drought and salt stresses. The transient expression of Gh_D12G2017-GFP fusion protein in the protoplast showed that *Gh_D12G2017* was localized in the nucleus. The transgenic Arabidopsis lines exhibited higher concentration levels of the antioxidant enzymes measured, including peroxidase (POD), superoxide dismutase (SOD), and catalase (CAT) concentrations under drought and salt stress conditions with very low levels of oxidants. Moreover, cell membrane stability (CMS), excised leaf water loss (ELWL), saturated leaf weight (SLW), and chlorophyll content measurements showed that the transgenic Arabidopsis lines were highly tolerant to either of the stress factors compared to their wild types. Moreover, the expression of the stress-related genes was also significantly up-regulated in *Gh_D12G2017* *(CDKF4)* transgenic Arabidopsis plants under drought and salt conditions. We infer that CDKF-4s and CDKG-2s might be the primary regulators of salt and drought responses in cotton.

## 1. Introduction

Cell multiplication is controlled by conserved molecular machinery in which the main players are the Serine/Threonine kinases, known as cyclin-dependent kinases (CDKs). CDK activity is regulated in a complex way, such as phosphorylation and or dephosphorylation by specific kinases in association with regulatory proteins [1]. CDKs are a large family of serine/threonine protein kinases that were first discovered for their role in regulating the cell cycle, they have diverse regulatory roles in a number of cellular and developmental processes in eukaryotes [2]. CDKs are also involved in mRNA processing, regulation of transcription, and also in the differentiation of nerve cells in animals [3]. They are found in all known eukaryotes, and their regulatory function in the cell cycle has been evolutionarily conserved, in plants, they are identified by both letters and numbers while in animals they are coded by numbers only [4]. 

A number of studies have linked *CDK* genes to drought and salt stress tolerance in addition to their primary role of regulating cell cycle processes [5]. Abiotic stresses including cold, drought, salt and heavy metals hampers plant growth and development and in turn significantly reduces plant productivity and yield. Overall drought and salt stress effects are estimated to be over 30% [6]. As a consequence of plant lifestyle, plants are immobile for most of their lifetime and, lacking mobility, they have to cope with wide changes in their ecological niches. Their successful adaptation to this sessile life-style can be attributed to their ability to adapt and respond to different types of biotic and abiotic stress. The cotton plant, also known as white gold, is the number one fiber producing plant and the main source of raw materials for the textile industries [7]. 

*Gossypium hirsutum*, is the main tetraploid cotton species cultivated for fiber and oil production across the globe, but its tolerance to drought stress is limited [8]. In order to maintain and increase the level of cotton production, new varieties with enhanced performance in the face of declining precipitation and or fresh water for agricultural production are inevitable [9]. Conventional breeding has yielded a limited extent and therefore the only sure mechanism to abate the threat imposed by drought is to explore molecular approaches in developing superior and stress tolerant cotton genotypes [10]. To this end, stress inducible genes have been identified and complex signaling cascades that mediate stress tolerance are currently being explored [11]. 

One of the major plant responses to moderating stress is the inhibition of cell proliferation and cell growth. While this response can lead to reduced yield in crops, it has a more general adaptive significance in that the resultant plant is more likely to survive under sudden or prolonged abiotic stress incidences [12]. Plant performance is directly proportional to the development of its various organs [13]. The development of plant organs directly depends on the frequency of cell division, parameters of the cell cycle, number, and size of the cells. These developmental processes are controlled by the molecular machinery that regulates cell cycle progression in coordination with nutritional, hormonal, developmental, and environmental factors [14]. The CDKs are core cell cycle regulators that are involved in various processes of plant growth and development [15]. 

A progressive transition through different phases of the cell cycle is controlled by a conserved mechanism based on the sequential formation and the activation of complexes between CDKs and their activating cyclin (CYC) subunits [16]. The *S. pombe SUC1* gene was identified in fusion yeast by its ability to rescue certain temperature-sensitive cdc2 mutants [17] and its homolog, the *S. cerevisiae CKS1* gene, was found to be a suppressor of *CDC28* mutants [18]. The *SUC1*/*CKS1* homolog was isolated from Arabidopsis using yeast two-hybrid screening and designated as *AtCKS1* [19]. The overexpression of *AtCKS1* in *A. thaliana* decreased the leaf size and reduced the root growth rate without any effect on endocycling [20]. Similarly, drought-stressed maize roots have been reported to display longer and relatively larger meristematic cells with a reduced zone of cell division, indicating a delay of the mitotic phase [21]. In wheat, the leaf mesophyll cell division has been reported to be reduced, a process which has been positively correlated with the inhibiting role of *CDKA1* activity [22]. The *CYCH;1* gene from Arabidopsis has been reported to be involved in drought stress response by regulating the blue light-mediated stomatal opening as well as controlling the reactive oxygen species (ROS) homeostasis [23]. In addition, the *CDKG2* gene has been found to confer tolerance to salt stress and promoting flowering in Arabidopsis [15]. In recent studies, Zhao et al. [24] showed that *CDK;2* increases both cell division and drought tolerance in Arabidopsis through the regulations of cycle genes and those involved in stomatal development. In light of the above, this gene family plays a significant role in plants in enhancing tolerance to various abiotic stress factors.

Given the potential roles of CDK proteins in the regulation of plant growth and development in response to environmental stresses, it is prudent to carry out a genome-wide analysis of this gene family in cotton, *Gossypium species*. Therefore, the objective of this study was to carry out a comprehensive analysis of the protein characteristics, gene classification, structure, chromosomal distribution, gene duplication, phylogenetic relationships, and functional analysis of cotton CDKs under salt and drought stress conditions through transforming *Gh_D12G2017*, one of the highly expressed genes, under drought and cold stress as determined through real time quantitative polymerase chain reaction (RT-qPCR). Our results will aid the understanding on the mechanism of cell cycle regulation and the functions of *CDK* genes in cotton under drought and salt stress condition.

## 2. Results

### 2.1. Identification and Sequence Analysis of CDK Proteins in Cotton Genome

To identify cyclin dependent kinase (*CDK*) genes in the three cotton genome, the conserved domain of CDK protein (PF00069) was downloaded from Pfam protein families (http://pfam.sanger.ac.uk/). The HHM profile of the CDK protein was subsequently employed as a query to perform a HMMER search (http://hmmer.janelia.org/) [25] against the *G. hirsutum*, *G. raimondii* and *G. arboreum*. A total of 31, 12 and 15 *CDK* genes were obtained for *G. hirsutum*, *G. arboreum* and *G. raimondii*, respectively (Table S1). 

The physicochemical parameters of each *CDK* gene were calculated by using an online tool, ExPASy (https://expasy.org/). All the CDKs in *G. hirsutum*, *G. arboreum* and *G. raimondii* had negative grand average of hydropathy (GRAVY) values ranging from −0.796 (*Cotton_A_13019*-*CDKC-2*) to −0.156 (*Gh_A03G1965*-*CDKF-4*), which implied that the cotton CDKs are hydrophobic, a property found to be common among the stress inductive genes, such as *LEA* genes. Cotton LEA2 has been found to have GRAVY values of less than 0 [26]. The molecular weights in kilodalton (kDa) ranged from 13.817 (*Gh_A09G0392*-*CDKB1-2*) to 91.144 (*Cotton_A_14138*-*CDKG-2*). The Isoelectric Point (pI) values ranged from 4.34 (*Gorai.001G006600*-*CDKF-1*) to 10.323 (*Cotton_A_25379*-*CDKF-4*). The CDKs were found to have low GRAVY values >0 and with high a charge ranging from −29.5 (*Gorai.001G006600*-*CDKF-1*) to 30.5 (*Cotton_A_25379*-*CDKF-4*), which is a unique feature also observed among the *CDK* genes in Arabidopsis [27]. Low hydrophobicity and high net charge are known to be the main characteristics of various stress inducible proteins, which enables them to be totally or partially disordered; this unique feature is an attribute which gives the CDK proteins the ability to form flexible structural elements such as molecular chaperones, which are integral for the protection of plants from desiccation effects [28]. 

The majority of the proteins encoding the *CDK* genes were found to be embedded within the cytoplasm. Among the proteins in *G. hirsutum*, 15 were located within the cytoplasm, accounting for 50% of all the CDK proteins in *G. hirsutum*, 11 (37%) in the nucleus, three in the chloroplast, while the plasma membrane had one. A similar prediction was evident among the proteins in the two diploid cotton species. In *G. arboreum*, high numbers of the proteins encoding the *CDK* genes were found to be cytoplasmic proteins (7), 4 were found in the nucleus, and only one was predicted to be located in the chloroplast. The same trend was also noted among the proteins encoding the *CDK* genes in *G. raimondii*, in which majority were predicted to be sub localized in the cytoplasm, nucleus and chloroplast with 9, 4, and 2, respectively. The result for the subcellular compartmentalization prediction of the proteins encoding the *CDK* genes were further validated through two online tools, TargetP1.1 (http://www.cbs.dtu.dk/services/TargetP/) server and Protein Pprowler Subcellular Localization Predictor version 1.2 (http://bioinf.scmb.uq.edu.au/Pprowler_webapp_1-2/). The results obtained were in agreement to those obtained from WoLF PSORT subcellular predictor online tool (Table S2)

### 2.2. Gene Structure and Amino Acid Motif Analysis of the CDK Genes in Cotton

**Figure 1:**
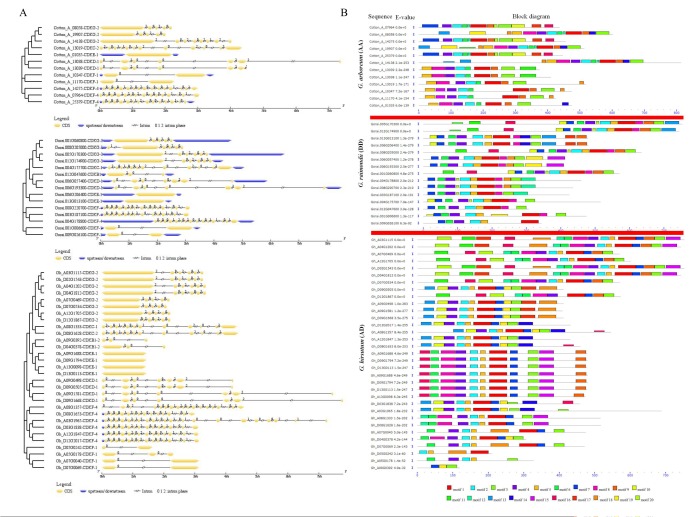
Gene structure and motif compositions of cotton *CDK* genes. (**A**) Exon/intron structures of *CDK* genes in cotton, exons introns and up/down-stream were represented by yellow boxes, black lines, and blue boxes, respectively. (**B**) MEME software characterized conserved motifs of *G. raimondii* (D), *G. hirsutum* (AD), and *G. arboreum* (A). Different colors represent different motifs and each motif is represented by a box numbered at the bottom. The names of genes and combined P value are exhibited on the left side of the figure. Grey lines represent the non-conserved sequences; the length of protein can be estimated using the scale at the bottom.

Gene structural diversity and conserved motif divergence are possible mechanisms for the evolution of multigene families [29]. To gain further information into the structural diversity of cotton *CDK* genes, we analyzed the exon/intron organization in the full-length cDNAs with their corresponding genomic DNA sequences of individual *CDK* genes in cotton. Most of the *CDK* gene members within the same class shared similar gene structures in terms of either intron numbers or exon lengths (Figure 1A). The highest intron disruption was noted in the members of the CDKF4s, with intron disruption ranging from 15 to 23. In general, all the sub families of the *CDK* genes in the three cotton species were disrupted with introns except for the CDKE, which were intronless. The results indicated the divergence functions of this protein family in cotton.

A motif is a consensus or a conserved region in the protein or nucleotide sequences. In the analysis of the motif, we identified 20 distinct motifs; however, motif 1 (WYRAPELLLGAKQYTSAVDMWSVGCIFAELLTLRPLFPGTSEIDQLGKIF), motif 2 (GLAYCH DNWVLHRDLKPSNLLV), and motif 4 (YLVFEYMEHDLYALMKDRKKKFSEVDIKC) were common in all the three cotton genomes, indicating that these conserved motifs may be playing key roles in subfamily-specific functions more so in stress related roles (Figure 1B and Table S3). The three common motifs were further compared to the already identified motifs by the use of the Tomtom motif comparison tool, adopting the distance measure of the Sandelin–Wasserman function [30]. Motif 1 had 3 matches, ELME000013, ELME000021, and ELME000389. Motif 2 with 8 matches, namely ELME00045; ELME000145; ELME000167; ELME000190; ELME000249; ELME000287; ELME000340, and ELME000422. Lastly, motif 4 had 9 matches, ELME000004; ELME000005; ELME000129; ELME000167; ELME000145; ELME000145; ELME000360; and ELME000424. With the MEME functional tool, we were able to affirm the similarities of our motifs to already published motifs in the motif database. Among different amino acids conserved in the 20 motifs, leucine (L) frequency was the highest (0.092), followed by Serine (0.074), Alanine (0.073), Glycine (0.069), Valine (0.064), Glutamic acid (0.062), and Threonine (0.059) etc. while the minimum frequency was the tryptophan (W) amino acid (0.013). All the 20, identified motifs were part of the protein kinase domain. The protein kinase domain has been found to play an important role in linking abiotic stress tolerance and the metabolic responses of plants [31].

### 2.3. Phylogenetic Analyses and Protein Alignments of the CDK-Proteins in Cotton with Other Plants

**Figure 2:**
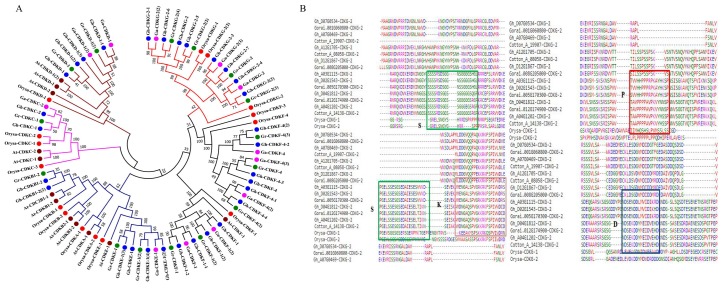
Phylogenetic tree and motif identification among the CDK proteins. (**A**) The neighbor-join phylogenetic tree of Cotton, Rice and Arabidopsis *CDK* genes. The red circle: CDKs from rice, blue: CDKs from *G. hirsutum*, green: CDKs from *G. raimondii*, brown: CDKs from Arabidopsis and purple: CDKs from *G. arboreum*. (**B**) Alignment of CDK motif domain. Color shading indicates types of amino acid residues conserved. S: motif, K: motif, P: motif and D: motif.

To get a better understanding of the evolutionary history and relationships of the *CDK* gene families in cotton to other plants, a multiple sequence alignment of 31 genes for *G. hirsutum*, 12 genes for *G. arboreum*, 15 genes for *G. raimondii*, 12 genes for Arabidopsis, and 15 genes for rice CDK protein sequences was carried out (Figure 2A). The reliability of the phylogenetic tree confirmed by reconstructing the phylogenetic tree adopting minimum evolution method, the two phylogenetic trees were identical, thus the results were consistent. Based on the Phylogenetic tree analysis and CDK annotation in Arabidopsis, the *CDK* genes were further classified into eight (7) groups, namely CDKA, CDKB, CDKC, CDKD, CDKE, CDKF, and CDKG, in which cotton CDKs were only found in 6 groups, no CDKAs was detected for any of the cotton genomes. The cladding pattern enabled us to identify ortholog genes, no ortholog genes was detected between any of the three cotton genome to the *CDK* genes in either Arabidopsis or rice genes. All the ortholog genes in the functional groups were found in *G. hirsutum* vs. *G. arboreum* and *G. hirsutum* vs. *G. raimondii.* More interesting, paralog genes were only found in Arabidopsis and rice. The presence of ortholog *CDK* genes in cotton, affirms the evolution of tetraploid cotton, *G. hirsutum*, which emerged due to a whole genome duplication of the A and D genome [32]. In the aligning of the protein sequences of *G. hirsutum, G. arboreum*, *G. raimondii*, *Oryza sativa*, and *Arabidopsis thaliana*, we identified motif D, E, G, L, P, Q, and R (Figure 2B). In the genome wide study of cyclin genes in Arabidopsis, the D domain has been identified, which is mainly found to be associated to A and B type cyclins, the D domain is also referred to as the destruction box (D-box), which is involved in cyclin proteolysis by the ubiquitin-dependent proteasome pathway. The identification of P,E,S confirms an earlier finding, which stated that the D-type cyclins have a motif called the PEST region, which is rich in Proline (P), Glutamic acid (E), Serine (S), and Threonine (T) residues, and is a marker for unstable proteins [33].

### 2.4. Chromosomal Distribution of Cotton Genes Encoding CDK Proteins

To determine the chromosomal locations of the cotton *CDK* genes based on their positions, data retrieved from the whole cotton genome sequences were used. Chromosome distribution was done by BLASTN search against *G. hirsutum* and *G. arboreum* in cotton genome project and *G. raimondii* genome database in Phytozome (http://www.phytozome.net/cotton.php). The upland cotton, *CDK* genes were not distributed in all the 26 chromosomes. The CDKs distributions between the two sub-genomes were equal with 14 genes each. The inability to find the *CDK* genes in all the chromosomes, could be a pointer to show that there could be an element of gene loss or these genes are chromosome specific, being all the homolog members of At chromosomes, at least the *CDK* genes were mapped onto their corresponding Dt chromosomes except for At-chr03, At-chr07, and At-chr13, which never had their corresponding homologous chromosomes in the Dt-sub genome. A similar pattern was also noted among the diploid cotton species (Table 1). Across the entire three genomes, A, D and AD, the sub class CDKD-1, had the highest distribution, and they were located in a common chromosome, for example 2 CDKD-1 in chromosome 11 (chr11) in A genome, 2 and 3 CDKD-1 in At-chr09 and Dt-chr09 in the AD genome, respectively, and for the D-genome, 3 CDKD-1 was mapped in chromosome 6 (chr06). This form of clustering pattern of the *CDK* genes and chromosomal position could be linked to the gene duplication pattern of the CDKs [34]. The CDKD-1 pattern of distribution in specific chromosomes and with high loci densities could be linked to ensuring maximum function and therefore CDKD-1 could be playing diverse roles in the plant to ensure its growth and survival under stress conditions [35]. 

### 2.5. Gene Duplication, Orthologs, Paralogs, and Selection Type of the CDK Genes

Gene duplication has been the main course of expansion of the various gene families in both plants and animals. The various forms of duplication are segmental, tandem, and whole genome duplication [36]. Homologous and orthologous genes are the products of gene duplication events. The multiplication of genes that retain the same identity has been associated to the stress related genes, and thus duplicated genes function in stress response and development processes in plants [37]. Most of the duplicated genes were detected between *G. hirsutum* and its ancestors, *G. arboreum* and *G. raimondii*, this could be due the origin of *G. hirsutum*, as a result of the whole genome duplication of the A and D genome (Table S4 and Figure S1).

Two types of duplication event, tandem and segmental duplication, were identified. The majority of the duplicated *CDK* genes were segmental as compared to the tandem type of duplication, this implied that segmental gene duplication had a major contributing factor during the evolution time [38]. The dS/dN ratio is a pointer to selective pressure acting on a protein-coding gene. It has been observed that some systematic bias in some species do occur more easily in the process of nucleotide substitutions because of species diversity and a high mutation rate accelerates the changes in amino acid proportions [39]. The analysis of the dS/dN ratios of the 120 paralogous gene pairs, 89 pairs had dS/dN ratios of less than 1, and only 31 had ratios of more than 1. The highest dS/dN ratio was of 7.2 was observed for gene pairs, *Gh_A13G0098*-*Cotton_A_01035*, which code for the CDKE-1 type of cyclin dependent kinase protein. 

In general, dS/dN ratios for the paralogous gene pair of entire cotton genomes *CDK* genes had a range of 0.206 to 7.1536 with a mean of 0.9752. This result gives an indication that the *CDK* genes have been majorly influenced by purifying the selection during the process of their evolution.

### 2.6. Promoter (Cis-Element) Analysis

**Figure 3:**
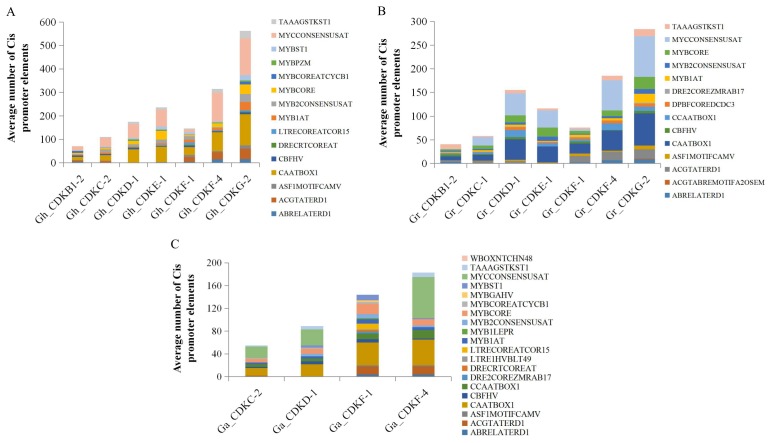
Average number of the cis-promoters ABRELATERD1 (ACGTG), DRECRTCOREAT (G/ACCGAC), MYBCORE (TAACTG), LTRE1HVBLT49 (CCGAC) and others in promoter region of the three cotton genomes (**A**) *Gossypium hirsutum*; (**B**) *Gossypium raimondii* and (**C**) *Gossypium arboreum*, *CDK* genes from each CDK subfamilies. The promoter regions were analyzed in the 1 kb upstream promoter region of translation start site using the PLACE database.

In order to determine the cis-acting regulator element, we queried a section of the sequence of each gene, but only the start and end codons were used for the selection of the cis-promoter elements. An analysis of the promoter region of the cotton *CDK* genes identified the presence of various stress responsive cis-acting elements, including DRE/CRT, ABRE, LTRE, and MYBS. These stress-responsive elements were relatively abundant in the promoter regions of the cotton *CDK* genes, more specifically ABREs (Figure 3A), indicating that CDK proteins may have an important functional role in drought stress response and tolerance in cotton. There were significant differences in the average proportions of the promoter elements detected within the different *CDK* gene sub groups (Figure 3B).

The cotton *CDK* gene types of CDKF-4 and CDKG-2 contained the highest number of cis promoter elements across the three cotton genome, maximum number achieved in CDKG-2 in *G. hirsutum* and *G. raimondii* while CDKF-4 was present in all with relatively high quantities of transcription factors related to stress such as MYBCORE (CNGTTR), MYB2CONSENSUSAT (YAACKG), ACGTATERD1 (ACGT), CAATBOX1 (CAAT), ABRELATERD1 (ACGTG) among others as illustrated in (Figure 3C). CAATBOX1, MYCCONSENSUSAT and ABRELATERD1 (ACGTG) were the abundant cis elements. Similar findings, with the predominance of ABRE cis-element, have been reported for *CDK* genes in tomato [40], Arabidopsis [41], and Chinese plum [42]. ABRE is a cis-acting element majorly involved in abscisic acid (ABA) signaling in response to abiotic stresses, while DRE/CRT and LTRE are major cis-acting regulatory elements involved in the ABA-independent gene expression in response to water deficit (DRE/CRT) and cold (DRE/CRT and LTRE) [43]. MYBs are known cis-acting promoter elements with critical role in the ABA-dependent signaling pathway in response to drought, salt, and cold [44].

### 2.7. RNA Sequence Expression Profiling of the CDK Genes under Drought and Salt Stress Condition

**Figure 4:**
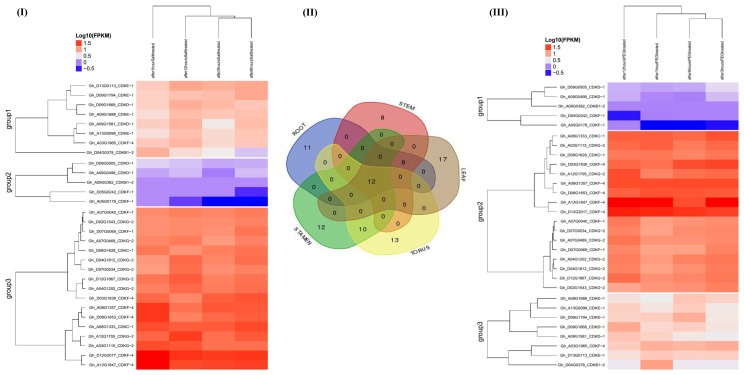
RNA sequence profiling of the *CDK* gene expression under salt and drought stress. (**I**) Heat map displaying expression changes of differentially expressed *CDK* genes in upland cotton, *G. hirsutum* plants stressed for 1 h, 3 h, 6 h, and 12 h of salt stress compared with the control; (**II**) Venn diagram depicting the expression of the *CDK* genes in various tissues and (**III**): Heat map displaying expression changes of differentially expressed *CDK* genes in upland cotton, *G. hirsutum* plant stressed for 1 h, 3 h, 6 h and 12 h of drought compared with the control.

The gene expression pattern can provide important clues for its function. To explore the expression pattern of the *CDK* gene family in cotton growth and development, we investigated the relative expression level of CDKs in five tissues, including root, leaf, stem, stamen, and torus. The results revealed that *CDK* genes have differential expression patterns in different tissues of cotton plant (Figure 4), and could be categorized into 3 groups (1 to 3) in relation to their tissue-specificity expression pattern. The 30 identified *CDK* genes in *G. hirsutum* showed differential expression (RPKM > 1) in the different tissues, under drought and salt stress condition, with exposure range from 1 h, 3 h, 6 h and 12 h. The abundance of the transcriptome factors was highly varied, the leaf expressed the highest amount followed by the torus, stamen, root and the least number was detected on the stem. Out of the total only 5 genes, such as *Gh_D09G0505* (CDKD-1); *Gh_09G0498* (CDKD-1), *Gh_A09G0392* (CDKF-1), *Gh_D05G0242* (CDKF-1), and *Gh_A05G0178* (CDK-1) were either down regulated or not expressed at all under drought and salt stress, an indication that these genes may not be directly involved in the two types of abiotic stress being investigated, furthermore, the same genes showed similar expression in the various tissues. Group 3 genes under salt stress profiling expressed the highest expression level, with 17 genes accounting for more than 50% of all the *CDK* genes present in *G. hirsutum.* Among the 17 highly expressed genes, two were significantly up regulated across the different stress exposure periods, from 1 h to 12 h, *Gh_D12G2017* and *Gh_A12G1847*, all the two were members of the CDKF-4 sub family, the same sub family was also found to harbor the highest level of the cis promoter elements across the three cotton genome. This could be a pointer of the significance role played by this sub family in plants under drought and salt stress.

### 2.8. RT-qPCR Analysis of the Cotton CDK Genes under Drought and Salt Stress

To examine the expression profile of CDK proteins family in various tissues under drought and salt stress treatments, we used all the genes as obtained for the three cotton genomes; 30 genes for *G. hirsutum,* 12 for *G. arboreum*, and 15 for *G. raimondii*. The main reason for carrying this detailed expression analysis was to examine the expression pattern of the ortholog genes as detected from the phylogenetic tree. Ortholog genes are genes that are of different genomes but are cladded together in the phylogenetic tree. In addition, being the evolution and expansion of these genes were found to occur through segmental duplication, the dS/dN ratios showed that the majority of the genes have been principally influenced by negative selection or purifying selection. It was our interest to look at the expression; if at all these genes had common or exhibited differential expression under drought and salt stress in the various plant tissues tested.

**Figure 5:**
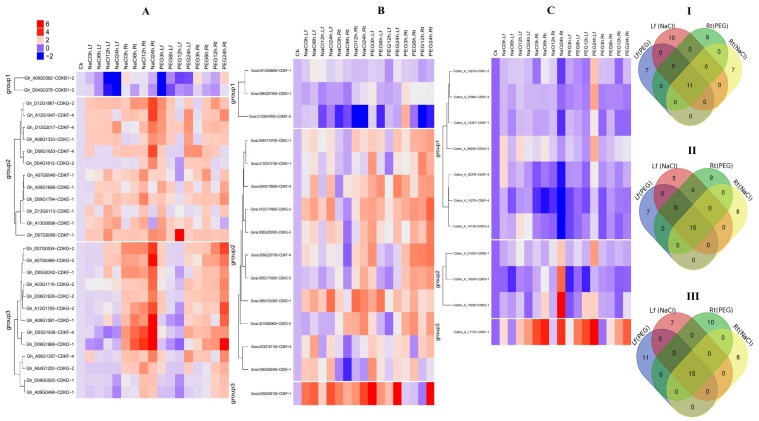
Transcriptome profiling of *CDK* gene expression through RT-qPCR under salt and drought stress. (**A**–**C**) Heat map displaying expression changes of differentially expressed *CDK* genes in *G. hirsutum*, *G. raimondii*, and *G. arboreum* respectively plants stressed for 3 h, 6 h, 12 h and 24 h of salt and drought stress compared with the control; (I, II and III) Venn diagram depicting the number of up regulated *CDK* genes in root and leaf tissues at 24 h of drought (PEG) and salt (NaCl) stress exposure.

Based on RNA expression, we found that majority of the genes were highly expressed, and therefore we validated the RNA sequence expression through qRT-PCR. Out of 30 CDK genes of *G. hirsutum*, 12 genes were found to have significant up regulation and were major members of cluster II (group 2). For instance, *Gh_D12G1867* (CDKG-2) and *Gh_A12G1847* (CDKF-4), showed the highest up regulation under salt stress at 24 h in the root tissues (Figure 5). This indicated that members of CDKGs and CDKFs have significant role under stress, more so under salinity stress. Roots are the very first plant organs to be into contact with high concentrations of salts in the soil [45]. The results obtained are consistent to previous findings in which the cyclin-dependent kinase regulatory subunit gene (CKS) was found to be highly up regulated in the roots and leaves of pigeon pea under drought and salt stress [46]. The up regulation of CDKGs and CDKFs in the roots could possibly be to maintain the root growth, to increase its capacity for water induction under salt and drought stress. It has been reported that the plants roots adapt to increased salinity levels through increased abundance of signal proteins, among which phosphoproteins with the role of activating the kinase cascade, which have been found to be highly up regulated in the roots of rice [47], maize [48], and creeping bent grass [49].

### 2.9. RT-qPCR Validation of the Highly Upregulated CDK Genes in Tisues of G. tomentosum (Tolerant) and G. hirsutum (Sensitive) to Drought and Salt Stresses 

**Figure 6:**
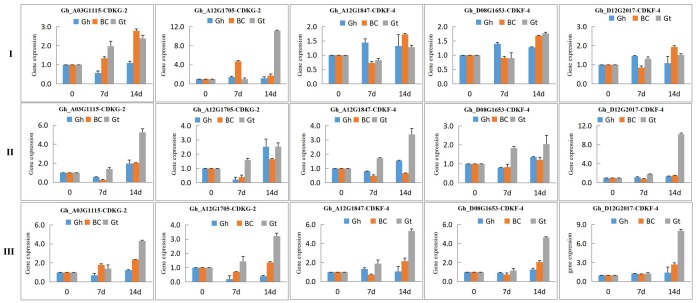
RT-qPCR expression profile of the top five highly expressed *CDK* genes in RNA sequences as compared to control with expression value 1.0 in upland cotton under drought stress. Gh: *Gossypium hirsutum*, BC: BC_2_F_1_ generation, Gt: *Gossypium tomentosum*. 0, 7 and 14 are days of drought stress exposure. **I**: Expression on leaf tissue; **II**: Expression on stem tissue and **III**: Expression on root tissue.

The five highly upregulated genes were further examined by carrying on expression analysis on two cotton species, *G. tomentosum* (tolerant) and *G. hirsutum* (sensitive) to both drought and cold stresses. All the genes showed significant up regulation across on the leaves, stem, and the roots at 0, 7, and 14 day of drought stress exposure (Figure 6). The results reaffirm the ability of the more tolerant genotypes capability of inducting more stress related genes during stress exposure. In the examination of ortholog genes, they exhibited differential expression, except for a few instances where they showed a similar expression pattern (Figure S2). The CDKGs and CDKFs are found to have a significant role under drought and salt stress in cotton, and therefore could be the possible putative genes responsible for enhancing tolerance to salt and drought stress.

### 2.10. Experimental Determination of the Subcellular Localization of Gh_D12G2017 (CDKF4) Protein

**Figure 7:**
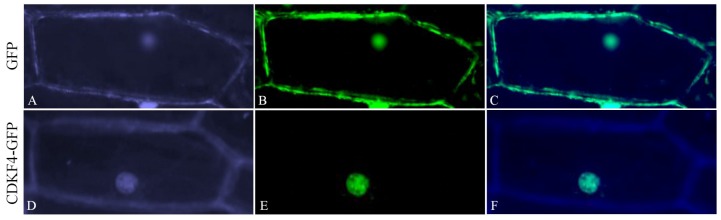
Localization of *Gh_D12G2017 (CDKF4)* in onion epidermal cells. (**A**–**C**) Onion epidermal cells transformed with 35S::GFP. (**D**–**F**) Onion epidermal cells transformed with 35S: Gh_D12G2017_*GFP* (**A**,**D**) Light field with magnification of X400 to display morphology. (**B**,**E**) Dark field images for the detection of green fluorescent protein (GFP) fluorescence. (**C**,**F**) Superimposed light and dark field images.

Based on bioinformatics analysis, an online tool wolfsport sub cellular localization tools showed that the protein encoded by *Gh_D12G2017 (CDKF4)* was predicted to be located within the nucleus. We sought to determine if the protein encoded by the transformed gene was embedded within the nucleus or not. We therefore made a *GFP-Gh_D12G2017 (CDKF4)*, fusion vector (pB1121-*Gh_D12G2017 (CDKF4)*, which was delivered into the onion epidermal cells by the 35S promoter through bombardment method [50]. The positive control showed that the gene was located within the nucleus (Figure 7). The detection of the transformed genes in the nucleus not only validate the Insilco analysis results but showed that proteins encoded by the transformed gene could be having a functional role in cellular regulation in relation to stress tolerance in plants. The nucleus is integral in the coordination of various cellular activities geared at improving the performance under abiotic stress conditions [51].

### 2.11. RT-qPCR Analysis of the Gh_D12G2017 (CDKF4) Gene in Upland Cotton Tissues and Confirmation of the Transfomed Lines of the Model Plant

**Figure 8:**
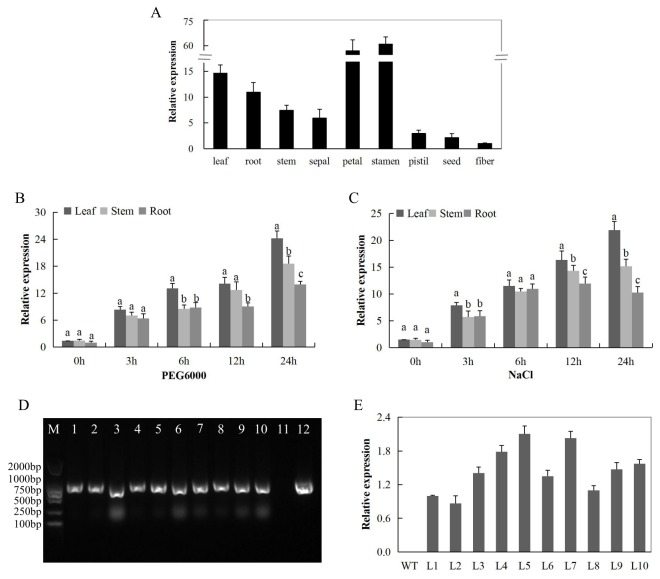
The RT-qPCR analysis of the expression of the cloned gene (**A**) Total RNA isolated from various tissue of cotton plant under normal conditions; (**B**) Total RNA extracted from drought-stressed cotton seedlings (**C**) Total RNA extracted from salt-stressed cotton seedlings; (**D**) Polymerase chain reaction (PCR) analysis performed to check 1299 bp coding sequence (CDS) integration in transformed T_1_ generation, number 1–10 transgenic Arabidopsis lines, 11 negative control (wild type) and 12 is the positive control (*pWM101-Gh_D12G2017 (CDKF4)* (**E**) The gene expression levels of the *Gh_D12G2017 (CDKF4)* of T_2_ transgenic Arabidopsis lines analyzed through RT-qPCR, in three biological replicates. Different letters (a, b, and c) indicate significant differences between expression levels of the gene in different tissues of the *Gh_D12G2017 (CDKF4)* overexpressed Arabidopsis lines (ANOVA; *p* < 0.05).

In order to evaluate the response of the novel gene in a cotton plant, we carried out a RT-qPCR analysis of the gene in various tissues of cotton plant; this was to determine the tissues with higher gene induction. In addition, the novel gene was also profiled in the cotton seedlings exposed to osmotic stress and salt stress through the use of PEG 6000 and a sodium chloride solution. The gene was abundantly expressed in the reproductive tissues, more specifically in the petal and stamen (Figure 8A). In addition, we carried out an expression analysis of the *Gh_D12G2017* gene in cotton seedlings under salt stress (250 mM NaCl) and osmotic stress (PEG6000_15%) conditions. Root, stem and leaf tissues obtained at 0, 3, 6, 12, and 24 h interval were analyzed. In salt and drought stress, the gene was highly abundant in the leaf tissues compared to the other two organs (Figure 8B,C). The expression detected for the transformed gene showed that the gene is present within the plant, and possibly among the genes with functional roles of enhancing abiotic stress tolerance in cotton. Ten lines were successfully transformed (Figure 8D), but only three lines, line 4, line 5 and line 7, were utilized in carrying out the functional analysis of the gene and they exhibited relatively higher expression of the gene compared to the rest of the lines (Figure 8E).

### 2.12. Response of the Overexpressed Lines and the Wild Type under Salt and Drought Stress Conditions

**Figure 9:**
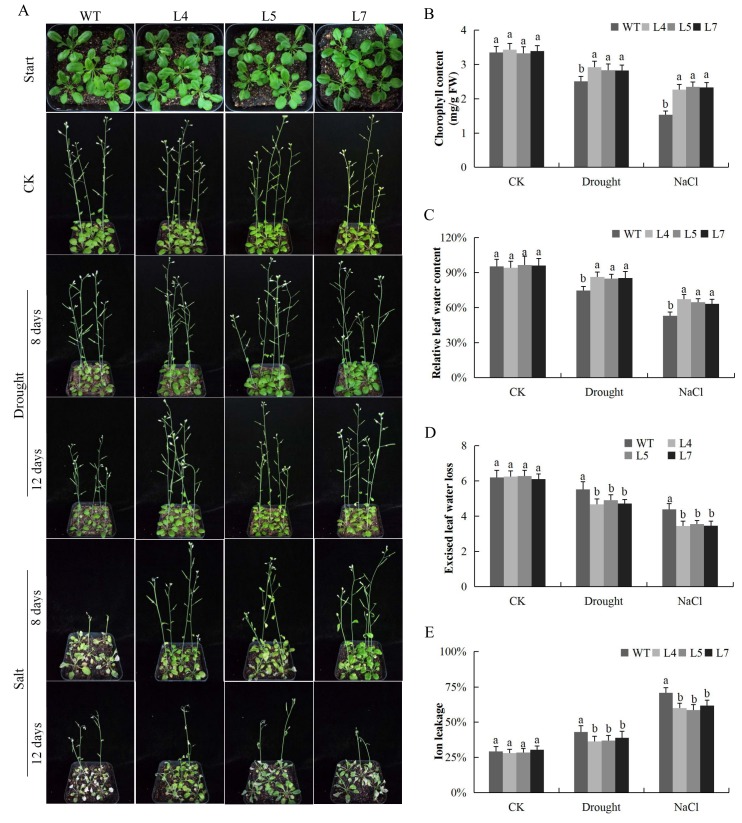
Determination of physiological traits (**A**) Transgenic Arabidopsis lines and wild type under abiotic stress conditions (**B**). chlorophyll content determination (**C**) Quantitative determination of relative water content (RLWC) (**D**) Quantitative determination of excised leaf water loss ELWL (**E**) Quantitative determination of cell membrane stability (CMS) as ion leakage concentration in leaves of wild type and transgenic Arabidopsis lines (L4, L5 and L7) after 8-day post stress exposure. Each experiment was repeated three times. Bar indicates standard error (SE). Different letters indicate significant differences between wild type and transgenic Arabidopsis lines (ANOVA; *p* < 0.05). CK: normal conditions.

**Figure 10:**
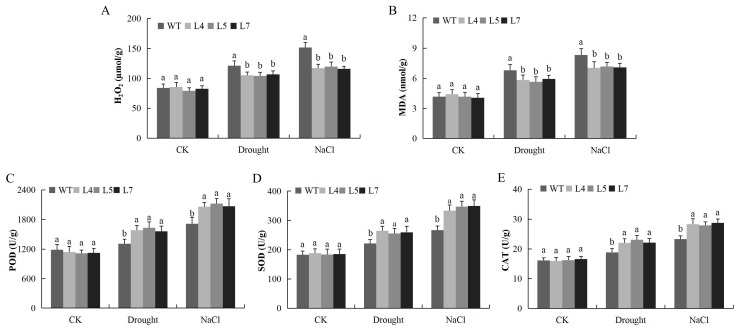
Determination of oxidant and antioxidant enzymes under drought and salt stress conditions (**A**) Quantitative determination of hydrogen peroxide (H_2_O_2_) concentration (**B**) Quantitative determination of malondialdehyde (MDA) concentration (**C**) Quantitative determination of POD concentration. (**D**) Quantitative determination of SOD concentration. (**E**) Quantitative determination of CAT in leaves of wild type and transgenic Arabidopsis lines (L4, L5 and L7) after 8-day post stress exposure. Each experiment was repeated three times. Bar indicates standard error (SE). Different letters indicate significant differences between wild type and the transgenic Arabidopsis lines (ANOVA; *p* < 0.05). CK: normal conditions.

The overexpressed lines exhibited higher tolerance to salinity and water deficit conditions. This was evident by the various physiological parameters, for instance, the chlorophyll content was significantly higher in the transgenic Arabidopsis lines under salt and drought stress conditions compared to the wild type (Figure 9A,B). In addition, relative water content on the leaves of the transgenic lines was higher compared to the wild type (Figure 9C). Similarly, in the evaluation of the excised leaf water loss on the two Arabidopsis plants, the transgenic and wild type, the transgenic Arabidopsis line exhibited lower levels of water loss compared to the wild type (Figure 9D). Moreover, cell membrane stability evaluated through ion leakage showed that the level of ion leakage was significantly reduced in the leaves of the transgenic Arabidopsis lines compared to the wild type (Figure 9E). CMS, ELWL, and SLW have been used extensively in screening for abiotic stress tolerance in major agricultural crops [52]. More significantly, the MDA level is a critical measure on lipid degradation due to the oxidation caused by excess reactive oxygen release and accumulation [53]. The MDA and H_2_O_2_ were significantly elevated in the leaves of the wild types as compared to the transgenic Arabidopsis lines, which showed significant reduction under similar conditions (Figure 10A,B). We further determined the levels of three reactive oxygen species scavenging enzymes, POD, SOD and CAT; the result showed that all the antioxidant enzymes were significantly higher in the leaves of the transgenic Arabidopsis lines compared to the wild types under salt and drought stress conditions (Figure 10C–E). The detection of higher concentrations levels of the various ROS scavenging enzymes showed that the overexpressed Arabidopsis lines were highly tolerant to oxidative stress, and thus minimized oxidative injury.

### 2.13. Evaluation of Stress Responsive Genes on the Tissues of Transgenic and Wild Arapidopsis Plants Under Salt and Drought Condition

**Figure 11:**
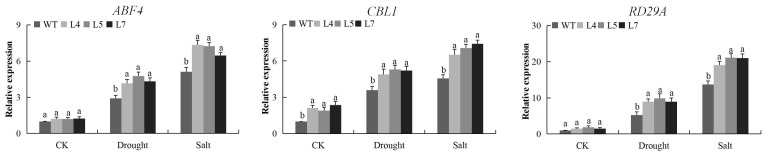
Expression levels of abiotic stress-responsive genes (*ABF4*, *CBL1* and *RD29B*) in transgenic Arabidopsis lines (L4, L5 and L7) and wild type Arabidopsis *Atactin2* gene was used as the reference, each experiment was repeated three times, mean values with ± SD, (a,b) calculated by Student’s *t*-test with *p* < 0.05.

The expression of three stress responsive genes known for abiotic stress tolerance, abscisic acid (ABA)-responsive element (ABRE)-binding transcription factors 4 (ABF4), responsive drought 29A (RD29A), and calcineurin B-like protein 1 (CBL1) was carried on the leaves obtained from the transgenic and wild type Arabidopsis plants. The result showed that the stress responsive genes were highly up-regulated in the transgenic Arabidopsis lines as opposed to the wild type (Figure 11A–C). The up regulation of the stress responsive genes showed that the cotton *CDK* gene, knocked in the model plant genome, had a functional role in enhancing the induction or mobilization of other plant transcription factors, to improve their tolerance towards drought and salt stress condition. The results obtained were in agreement with our previous work, in which the G-protein coupled receptor (GPCR) overexpressed lines and showed higher induction of the stress responsive genes compared to the wild type [54]. In addition, more stress tolerant plants have been found to show higher capability to induct more genes compared to the sensitive ones, in the analysis of the *LEA* genes on two cotton species, Magwanga et al. [55], found that the drought tolerant cultivar showed more up regulated genes compared to the drought susceptible cultivar.

## 3. Discussion

The function of *CDK* genes has been widely investigated in many plants species, but to date, no detailed analysis of the *CDK* gene family members has been reported in cotton and therefore their functions are still obscure. In this study, we identified 57 *CDK* genes in three cotton species, 31, 15, and 12 genes in *G. hirsutum*, *G. raimondii* and *G. arboreum*, respectively. The number identified in upland cotton, *G. hirsutum* was relatively higher than in *G. arboreum* and *G. raimondii*, this could be due to whole genome duplication (WGD), being tetraploid cotton emerged from the polyploidization of the A and D genomes [56]. In addition, we identified 11 and 15 *CDK* genes for rice and Arabidopsis, respectively. The results were in agreement to previous findings in which only 12 *CDK* genes were among the 90 putative core cell cycle proteins identified in rice [57]. The high number of *CDK* genes in *G. hirsutum* was more likely caused by gene duplication and the conservation of the *CDK* genes during the polyploidization process, indicating the significant role played by these groups of gene families in the process of plant growth and development [41].

As a result of the selection pressure imposed by extreme and variable environmental conditions, stress regulatory response and sensing mechanisms in plants have undergone a drastic transformation. One of the significant sources of plant innovation to stress response mechanisms is gene duplication [58]. Gene duplication is a major feature of genomic architecture, with a cardinal role in the process of plant genomic and organismal evolution, resulting in new raw genetic materials for genetic drift, mutation and selection, which ultimately results in the emergence of new gene functions and evolution of gene networks [59]. Gene duplication mechanism not only lead to the expansion of genome content but aids in the diversification of gene function to ensure adequate adaptability and the evolution of plants [36]. Therefore, the ability of the plants to sense and respond properly to abiotic cues, such as salt, drought, chilling and others is critical for plant survival [60]. In this study, the majority of the duplicated were found to be the segmental type, with 98 paralogous pairs accounting for 49 genes out of the 60 duplicated genes. Though, despite the abundance of segmental duplicated genes, we observed 28 paralogous gene pairs to have undergone tandem duplication, translating to only 11 genes out of the 60 duplicated ones. Segmental duplication is found to be dominant over the tandem in the process of gene evolution. For instance, the complete sequencing of the *A. thaliana* genome revealed numerous large-scale segmental duplications as opposed to tandem duplication [61]. The results obtained for cotton CDKs in which the segmental type of duplication was highly favored as opposed to the tandem is consistent with the previous results obtained in the analysis of cyclin dependent kinases in Arabidopsis, in which 22 core cell cycle genes were found to have emerged through segmental duplications [62]. The role of segmental duplicated genes in relation to drought have been reported, for example, the expansion of *Hsf* genes in sesame was found to have occurred through segmental duplication, and it is known to confer drought tolerance sesame [63].

Phylogenetic tree analyses have been vital in revealing the patterns of evolution of many genes. However, the importance of phylogeny reconstruction applies not only to the organisms that house the genes but also to the evolutionary history of the genes themselves. A phylogenetic analysis provided evidence of the contribution of whole genome duplication on the cotton *CDK* genes abundance as compared to its parental lineage, the diploid cotton, A and D genome. The *G. arboreum* of A genome had 12 *CDK* genes while *G. raimondii* of D genome with 15 *CDK* genes; therefore, the high number of *CDK* genes in *G. hirsutum* was due to the whole genome duplication (WGD). The expansion of *CDK* genes majorly occurred through segmental duplication but with restricted distribution across the whole cotton genome, being some of the chromosomes were found to lack the genes. This type of expansion is attributed to large segmental duplications that originated from continuous polyploidy events and has been subjected to scrambling by chromosomal rearrangements; the same has been observed for *CDK* genes in Arabidopsis [64]. The *CDK* genes were mainly found in only 16 chromosomes in tetraploid cotton, 9 and 10 in *G. arboreum* and *G. raimondii*, respectively. The results were consistent to previous report in which the *CDK* genes distribution were found to have restricted distribution and not found across the whole genome, for instance, the cyclin genes were found to be distributed in all the 11 chromosomes in tomato except for chromosome 8 [65].

Characterization and structural analysis of genes with major functions on abiotic and biotic stress factors have been found to have fewer introns [66]. The structural analysis of the *CDK* genes in cotton revealed a variation in terms of the intron-exon ratios, the CDKE-1 were all intronless except for one. The remaining genes were either disrupted by one or more introns. But it is important to note that even though introns possess a burden of the functionality of the genes, because of the demand for spliceosome, which is among the largest molecular complexes in the cell, comprising 5 small nuclear RNAs and more than 150 proteins [67], CDKF-4 had the highest disruption with exon numbers ranging from 15 to 24, but from the RNA sequence profiling of various tissues under drought and salt, the CDKF-4 sub family were found to be highly up regulated and could possibly be the putative genes conferring drought and salt stress tolerance in cotton. This finding contradicts the known facts of the effects introns has on gene function. The CDKF-4 sub family of the *CDK* genes had intron-exon lengths ratios ranging from 0.5262 to 1.2416 in the base pair. Similarly, the CDKG-2 had intron-exon lengths ratio ranging from 1.346 to 2.916 in base pairs. These two sub families were significantly up regulated under drought and salt stress, a pointer to indicate that not only the number of introns which determine the functionality of the genes but also the intron-exon lengths ratios. The variation in intron-exon lengths has been reported in other stress related genes such as *Hsf* genes [63].

The motif protein analysis and composition of each *CDK* genes sub family largely varied, although some amino acid-rich regions were detected, similar to previous studies done on Arabidopsis [27] and tomato [65]. Through meme and alignment, we found that that genes belonging to the same families exhibited similar gene structure and motif composition. This result is consistent to previous studies, which recorded similar exon-intron and protein motif within the same group of the *CDK* genes in the genome wide studies in tomato [65]. In this study, we idetified new motifs, which we denoted as motif D and K, which has never been recorded. Other motifs identified were P and S. All the motifs were common in *arapidopsis*, rice, *G. hirsutum*, *G. arboreum,* and *G.raimondii*. These motifs could help in functional characterizations of *CDK* genes in cotton.

The majority of the genes showed significant up regulation under salt and drought stress conditions. A unique observation was made on the expression pattern of the ortholog genes, being ortholog genes are of common origin, their expression pattern was not common across the tissues tested under drought and salt stress. For instance, *Gh_A04G1202* (CDKG-2) and *Cotton_A_14138* (CDKG-2) showed differential expressions across the time scale and in the tissues tested. This is an indicator of the orthologs genes acquiring new functions over time after evolution. The findings were consistent to the results obtained in the analysis of the expression levels of orthologs genes in *Glycin max,* in which the expression levels of the orthologs genes were found to show variation under drought stress [68]. In addition, the expression profiling of the highly up regulated genes in two cotton species with varying stress tolerance, the genes were highly up regulated in the tolerant cultivar compared to the sensitive genotypes. The results showed that the tolerant cultivar has a higher capacity to induct more genes, similar results have also been reported in maize [55].

In the evaluation of the transgenic Arabidopsis lines and their wild type under drought and salt stress condition, the transgenic Arabidopsis lines were found to have higher levels of antioxidant enzyme mobilization compared to their wild types under stress conditions. When plants are exposed to any form of stress, the normal and delicate balance of reactive oxygen species (ROS) release and detoxification becomes altered, leading to the over-production and accumulation of ROS, which causes oxidative damage to the cells [69]. The increased levels of the various antioxidants showed that the transgenic Arabidopsis plants had the capacity to tolerate the effects caused by salt and drought stress. Moreover, the transgenic Arabidopsis lines exhibited stable physiological and morphological traits associated with tolerance such has CMS, ELWL and SLW. The level of CMS in the leaves of the transgenic Arabidopsis lines as evaluated through ion leakage showed that the transgenic Arabidopsis lines had minimal oxidative injuries under salt and drought stress, due to very low ion leakage. Increased levels of ROS in the cell leads to membrane damage and even cell death [70]. Thus, the ROS are scavenged by the antioxidant enzymes, thereby reducing their deleterious effects in the plants during stress condition.

The stress responsive genes were found to be highly up regulated in the transgenic Arabidopsis lines, RD29A, CBL1, and ABF4 were highly induced in the tissues of the transgenic Arabidopsis lines. *RD29A* and *RD29B* gene have been intensively investigated and found to have a high potential in enhancing various abiotic stress tolerance in plants [71]. The overexpression of these stress responsive genes in the *Gh_D12G2017 (CDKF4)* overexpressed Arabidopsis lines indicated that the CDKs are not only important in cell regulation but also play a critical role in enhancing stress tolerance.

## 4. Materials and Methods

### 4.1. Identification and Sequence Analysis of CDK Proteins in Cotton Genome

The conserved domain of CDK protein domain (PF00069) was downloaded from Pfam protein families (http://pfam.sanger.ac.uk/). In order to identify the CDK proteins in cotton, the HHM profile of CDK protein was employed as query to perform a HMMER search (http://hmmer.janelia.org/) against the *G. hirsutum*, *G. raimondii* and *G. arboreum*. The CDK protein for *G. hirsutum* obtained from cotton research institute (http://mascotton.njau.edu.cn), *G.arboreum* obtained from Beijing genome institute (https://www.bgi.com/) and *G. raimondii* genome and *A. thaliana* downloaded from Phytozome (http://www.phytozome.net/), with E-value < 0.01. All redundant sequences were discarded from further analysis based on cluster W^17^ alignment results. To confirm the presence of CDK domain, the PROSITE (http://prosite.expasy.org/scan prosite/) and the SMART program (http://smart.embl-heidelberg.de/) were used for further analysis. SMART and PFAM database were used to verify the presence of the *CDK* gene domains. The isoelectric points and molecular weights of CDK proteins were estimated by ExPASy Server tool (http://www.web.xpasy.org/compute_pi/). In addition, subcellular location prediction of all the CDKs was conducted using the TargetP1.1 (http://www.cbs.dtu.dk/services/TargetP/) server and Protein Prowler Subcellular Localisation Predictor version 1.2 (http://www.bioinf.scmb.uq.edu.au/pprowler_webapp_1-2/). Validation and determination of the possible cell compartmentalization of all the CDK proteins was done by WoLF PSORT (https://www.Wolfpsort.hgc.jp/) [72].

### 4.2. Chromosomal Location and Gene Duplication of CDK Genes in Upland Cotton

The chromosomal distribution of *CDK* genes were mapped on cotton chromosomes based on gene position using mapchart 2.2 software [73]. Homologous genes of *G. hirsutum*, *G. raimondii* and *G. arboreum* were identified by BLASTP with threshold >80% in similarity and at least in 80% alignment ratio to their protein total lengths. Default parameters were maintained in all of the steps. Tandem duplications were designated as multiple genes of one family located within the same or neighboring intergenic region [74].

### 4.3. Phylogenetic Analysis, Gene Structure, Motif and Functional Classification of CDK Proteins in Cotton

Full-length sequences of *G. hirsutum*, *G. arboreum*, *G. raimondii*, *Oryza sativa* and *A. thaliana* CDK proteins were first aligned using ClustalW, then used MEGA 6 to conduct phylogenetic analyses based on protein sequences, with the neighbor joining (NJ) method [75]. Support for each node was tested with 1000 bootstrap replicates. The gene structures were obtained through comparing the genomic sequences and their predicted coding sequences from the cotton genome project. To identify the orthologous genes in cotton, *G. hirsutum* CDKs, were subjected through a BLASTp search of their protein sequences against the protein database of *G. arboreum*; *G. raimondii* and *A. thaliana*; hits with E-values ≤ 1 × 10^−40^ and at least 90% similarity were considered significant. The CDKs in Arabidopsis was obtained from TAIR (https://www.arabidopsis.org/browse/genefamily/CDPK.jsp), the sequences obtained were later confirmed through SMART search as describe in cotton CDKs identification. The CDKs proteins in rice were searched by using FGP-MINE Programs on the Floral Genome Project Website (http://www.fgp.bio.psu.edu/cgi-bin/fgpmine/fgp_family_list.cgi). To identify other CDKs proteins in rice, BLAST searches and sequence alignments were performed against the KOME (http://www.cdna01.dna.affrc.go.jp/cDNA/) and NCBI (http://www.ncbi.nlm.nih.gov/) databases. We employed BLASTP program in NCBI and KOME against the Arabidopsis CDKs proteins, with the E-value cut-off set as 1e-005. Gene structures of the three cotton genome *CDK* genes were constructed using a gene structure display server (http://gsds.cbi.pku.edu.cn). The protein sequences were analyzed for motif identification by MEME program. The MEME program was employed using the following parameters: number of repetitions-zero or one, maximum number of motifs 20, optimum motif width set to >6 and <50.

### 4.4. Signal Peptide and Promoter Cis-Element Analysis of the Cotton CDK Genes

The signal peptides of cotton CDKs proteins were predicted using SignalP (http://www.cbs.dtu.dk/services/SignalP/) with a D-cut-off value >0.5 as described by Magwanga et al. [26]. The 1500 bp upstream sequences before the translation initiation codon ATG of each cotton *CDK* genes were selected as gene promoters. Cis-regulatory elements of each promoter sequences were predicted though searching the PlantCARE database (http://bioinformatics.psb.ugent.be/webtools/plantcare/html/), the validation of the promoters were later done by using the PLACE database (http://www.dna. affrc.go.jp/PLACE/ signalscan.html).

### 4.5. Analysis of the Expression Patterns of Cotton CDK Genes in Different Tissues under Drought Stress Using RNA-seq Data

In order to determine the expression profile of upland cotton, *G. hirsutum CDK* genes in various tissues, we obtained RNA-seq data from root, stem, seed, torus and leaf, downloaded from cotton research institute data base (http://mascotton.njau.edu.cn). The read per kilo base per million mapped reads (RPKM) values were log10 transformed and used for the construction of the heat map with the R Software [76].

### 4.6. Plant Materials and Stress Treatments

Healthy Seeds of *G. hirsutum*, *G. raimondii* and *G. arboreum* obtained from the Institute of cotton research, Chinese Academy of Agricultural Sciences (CAAS), Henan, China, were delinted, pre-treated and germinated on wet filter paper for 3 days at 25 °C before being transferred to hydroponic setup with Hoagland nutrient solution [77], in a greenhouse with conditions set at 28 °C day/25 °C night, 14 h photoperiod, 60–70% relative humidity. The seedlings at the three true leaves stage were subjected to drought and salt stress by transferring them to nutrient solutions with 250 mM sodium chloride (NaCl) and 15% of PEG-6000, respectively. Roots and leaves were collected from the seedlings at 3 h, 6 h, 12 h and 24 h post-treatment. Untreated plants served as controls. Each treatment was repeated three times. For each biological replicate, the roots and leaves were collected from two individual seedlings. The samples upon collection were immediately frozen in liquid nitrogen and stored at −80 °C for RNA isolation.

### 4.7. RNA Isolation and RT-qPCR analysis

RNA extraction kit, EASYspin plus plant RNA kit, obtained from Aid Lab, was used to extract total RNA from roots and leaves. The quality and concentration of each RNA sample was determined using gel electrophoresis and a NanoDrop 2000 spectrophotometer, only RNAs which met the criterion 260/280 ratio of 1.8–2.1, 260/230 ratio ≥ 2.0, were used for further analyses and stored at −80 °C. The cotton constitutive *Actin7* gene was used as a reference and specific *CDK* genes primers were used for qRT-PCR. The first-strand cDNA synthesis was carried out with TranScript-All-in-One First-Strand cDNA Synthesis SuperMix for qRT-PCR, obtained from TRAN, it was used in accordance with the manufacturer’s instructions. Primer 5 was used to design *CDK* gene specific primers, with melting temperatures of 55–60 °C, primer lengths of 18–25 bp, and amplicon lengths of 101–221 bp.

Details of the primers are shown in Table S5. Fast Start Universal SYBR green Master (Rox) (Roche, Mannheim, Germany) was used to perform RT-qPCR in accordance with the manufacturer’s instructions. Reactions were prepared in a total volume of 20 μL, containing 10 μL of SYBR green master mix, 2 μL of cDNA template, 6 μL of deionised H_2_O and 2 μL of each primer to make a final concentration of 10 μM. The PCR thermal cycling conditions were as follows: 95 °C for 10 min; 40 cycles of 95 °C for 5 s, 60 °C for 30 s and 72 °C for 30 s. Data were collected during the extension step: 95 °C for 15 s, 60 °C for 1 min, 95 °C for 30 s and 60 °C for 15 s. Three biological replicates and three technical replicates were performed per cDNA sample.

### 4.8. Validation of the Ortholog and Key CDK Genes in Cotton under Drought Stress

*G. hirsutum* and *G. tomentosum* tissues were used. The two upland cotton accessions are perennially grown and maintained by our research group, in Sanya Island, Hainan province, China. The seeds were sown and grown under greenhouse condition, using soil as the rooting medium. Soil moisture content was monitored daily by use of the soil moisture meter, Em50, DECAGON. Upon emergence of the third true leaves, watering was totally withdrawn to impose drought stress. For drought treatment, soil was weighed before sowing to determine the amount of water in the soil at the beginning of the experiment. Controlled plants, soil moisture content was maintained by giving plants water to keep the soil moisture level at 30% of field capacity, which is 200% or 2 g water per 1 g of dry soil. The soil is known to be well watered when the soil water potential is above −30 kPa [78]. The leaf, root and stem tissue samples were then collected at 0, 7 and 14 days of drought stress exposure for RNA extraction and subsequent RT-qPCR analysis. *G. hirsutum* is an elite cultivar and predominantly cultivated for fiber and oil production in major cotton producing countries, though susceptible to drought and salt stress. *G. tomentosum* is a wild cultivar, known for its resistance to drought and salt stress due to its ecological niche, endemic to the Hawaiian island.

### 4.9. Clonining of the Highly Expressed Gene under Salt and Drought Stress, Gh_D12G2017 (CDKF4) in Arabidopsis thaliana (Ecotype Colombia-0) Lines

Cloning experiments were conducted using the *Escheria coli* strain (DH5α). Overexpression of the recombinant proteins was conducted using the *E. coli* strain BL21 (DE3). The transient expression of the protein in *Arabidopsis thaliana* was performed using *A. thaliana* ecotype Colombia-0 (Col-0). The *Agrobacterium tumefaciens* GV3101 floral dip method was applied by transforming the *A. thaliana* wild type [79]. The pWM101-35S:*Gh_D12G2017 (CDKF4)* construct in A. tumefaciens GV3101 was confirmed by gene specific primer, *Gh_D12G2017* _F (5′-CGGATCCATGGAGAGGTACAAGTTACTC-3′) and *Gh_D12G2017* _R (5′-GGTCGACTTAGAATCCGCAGTCCACAGA-3′). Host cells were grown in LB solution composed of 4.3 g/L, sucrose 50 g/L (5%), 2-(4-morpholino) ethane sulfonic acid (MES) 0.5 g/L, Silwet-77 200 µL/L (0.02%), 6-benzylaminopurine (6-BA) 0.01 mg/L with pH of 5.7. Transgenic Arabidopsis lines of *A. thaliana* were selected by germinating seeds on 50% (0.5) MS (PhytoTechnology Laboratories, Lenexa, KS, USA), containing 50 mg/L hygromycin B (Roche Diagnostics GmbH, Mannheim, Germany) for a duration of three (3) days before being transferred into a well-conditioned room, for the generation of T_0_. The T_0_ generated the T_1_ lines, and the selection done by germinating in 0.5 MS media in selective antibiotics. Only those with a germination ratio of 3:1 were selected for the generation of T_2_. The T_2_ were subsequently grown in selective antibiotics, and only those with 100% germination were used to generate the T3, which were subsequently used for functional analysis under salt and drought stress conditions, after evaluation through RT-qPCR, *Gh_D12g2017* gene specific primer (Forward seq 5′-TTATGTTCCGCCCTCTCTTCGTC-3′ and the reverse sequence 5′-TGTTCCCATGTTCCTTTCACCCC-3′) for the overexpressed lines.

### 4.10. Response of Overexpressed Gh_D12G2017 (CDKF4) Transgenic Arabidopsis Lines to Drought and Salt Stress Condition

After 7 days of growth in 0.5 MS, the *Gh_D12G2017 (CDKF4)* overexpressed lines and the wild seedlings were transplanted into small boxes filled with vermiculite and humus mixed in equal measure. After 21 days of growth, the plants were subjected to drought and salt stress treatment, by irrigating the plants with water containing 0 mM NaCl, 150 mM NaCl, and 250 mM NaCl for salt stress condition while for those under drought treatment, watering was totally withdrawn. Evaluation of moisture and water potential was determined at the onset of the experiment as described in Section 4.9. Evaluation for the oxidants and antioxidant enzymes was carried out after 8 days post stress exposure. Catalase (CAT), superoxide dismutase (SOD) and peroxidase (POD) were the antioxidant enzymes evaluated, while for oxidants, Malondialdehyde (MDA) and hydrogen peroxide (H_2_O_2_) were quantified. The procedure was described by Magwanga et al. [26]. In addition to biochemical evaluation, various cell physiological parameters were also evaluated, including chlorophyll content, cell membrane stability (CMS), excised leaf water loss (ELWL) and saturated leaf weight (SLW).

### 4.11. RT-qPCR Analysis of the Expression of Abiotics Stress Responsive Genes in Transgenic and Wild Type Lines

The seeds of the wild types and the transgenic Arabidopsis lines were sterilized and plated in 0.5 MS for 7 days, then transferred to small pots filled as described in Section 4.11 above. Drought and salt stress was imposed by irrigating with 250 mM of NaCl solution while for drought stress, no water was applied. Samples for RNA extraction were taken after 4 days post stress exposure. EASYspin Plus plant RNA extraction kit was used prescribed within the manual. Gel electrophoresis and a NanoDrop 2000 was used to evaluate the quality and concentration of the RNA extracted. The extracted RNAs were reverse-transcribed into cDNAs and used as templates for the PCR analysis. The specific primers for the stress responsive genes (*ABF4*, *RD29A* and *CBL1*) together with Arabidopsis *Actin2* gene were applied as the reference gene (Table S6). The RT-qPCR reaction samples and conditions were done as described in Section 4.8.

### 4.12. Experimental Determination of the Subcellular Location of the Transformed Gene by Use of Onion Epidermal Cell

The open reading frame of *Gh_D12G2017 (CDKF4)* was amplified by polymerase chain reaction using the transformed gene specific primer. The forward sequence *Gh_D12G2017 (CDKF4)* 5′-ACACGGGGGACTCTAGAGGATCCATGGAGAGGTACAAGTTACT-3′ and reverse sequence *Gh_D12G2017 (CDKF4)* 5′-ACTCATACTAGTCCCGGGGATCCGAATCCGCAGTCCACAGAAC-3′ was synthesized by Invitrogen, Beijing, China, and the *Pfu* DNA polymerase was obtained from the TransGen Biotech, Beijing, China. The polymerase chain reaction products were then transformed into a plasmid, pBI121-GFP vector upstream of the green fluorescent protein (GFP) to give pBI121- *Gh_D12G2017 (CDKF4)*–GFP construct with *Gh_D12G2017 (CDKF4)–GFP* fusion gene under the regulation of CaMV 35S promoter. The cloning process was done as per the kit manual, pEASY-Uni Seamless Cloning and Assembly Kit, which was obtained from the TransGen Biotech, Beijing, China [80]. The construct was then transferred into *A. tumefaciens* strain LBA4404, which was obtained from the Shanghai Weidi Biotechnology Co., Shanghai, China, and transformed into epidermal cells isolated from the onion bulb. Using the method of Sun et al. [81]. The transformed onion epidermal cells were cultured in 50% Murashige and Skoog (MS) media in a dark growth chamber for 20 h at 25 °C. The expression of the gene transformed into the onion epidermal cells was observed using a Zeiss Model Axio Imager M1 Upright Fluorescent Microscope (430004-9901-Axio Imager.M1, Gottingen, Germany).

## 5. Conclusions

In this research, we identified 31, 12 and 15 proteins encoding the CDK genes in *G. hirsutum*, *G*, *arboreum* and *G. raimondii*, respectively. The cotton *CDK* genes were found to primarily evolve through a segmental type of gene duplication as opposed to tandem. The identification of the various proteins encoding *CDK* genes in the three cotton and functional characterization has revealed their potential role in cotton under abiotic stress conditions. The *Gh_D12G2017 (CDKF4)* gene overexpressed plants showed higher capacity to tolerate drought and salt stresses as evident by higher biomass accumulation, increased elevation of the antioxidants, and a reduction in oxidants when exposed to drought and salt stress. The antioxidants evaluated in the tissues of the *Gh_D12G2017 (CDKF4)* gene overexpressed plants under salt and drought, such as POD, SOD, and CAT were detected in higher concentrations compared with the wild types. The main role of the antioxidants is to scavenge on the excessed produced ROS, thereby detoxifying the ROS [82]. Moreover, CMS, ELWL, and SLW traits which have been used extensively in screening for abiotic stress tolerance in various plants [83], indicating that the *Gh_D12G2017 (CDKF4)* overexpressed Arabidopsis lines were much more tolerant to drought and salt stresses compared to the wild types. In conclusion, the results of this research work provide a better opportunity for the utilization of the *CDK* genes in enhancing drought and drought tolerance in elite cotton cultivars. Being drought and salt stresses are the major abiotic stress with profound effects in the production of cotton globally.

## Figures and Tables

**Table 1:** Identification and chromosomal distribution of the cotton *CDK* genes.

Cotton Species	Gene ID	Gene Name	Description	Chro.	Start	End	Strand	Length (bp)
*G. hirsutum (AD*)	Gh_A09G0392	CDKB1-2	Cyclin-dependent kinase B1-2	A09	25,358,935	25,360,412	+	1478
	Gh_D04G0378	CDKB1-2	Cyclin-dependent kinase B1-2	D04	5,886,575	5,888,627	+	2053
	Gh_Sca005019G02	CDKB1-2	Cyclin-dependent kinase B1-2	scaffold	8,409	9,886	−	1478
	Gh_A08G1333	CDKC-1	Cyclin-dependent kinase C-1	A08	86,315,556	86,319,915	−	4360
	Gh_D08G1628	CDKC-2	Cyclin-dependent kinase C-2	D08	51,109,124	51,113,530	−	4407
	Gh_A09G0498	CDKD-1	Cyclin-dependent kinase D-1	A09	39,565,295	39,569,539	−	4245
	Gh_A09G1581	CDKD-1	Cyclin-dependent kinase D-1	A09	69,169,679	69,177,142	+	7464
	Gh_D09G0505	CDKD-1	Cyclin-dependent kinase D-1	D09	24,483,522	24,487,751	−	4230
	Gh_D09G1668	CDKD-1	Cyclin-dependent kinase D-1	D09	44,329,951	44,337,731	+	7781
	Gh_A09G1688	CDKE-1	Cyclin-dependent kinase E-1	A09	70,391,997	70,393,439	+	1443
	Gh_A13G0098	CDKE-1	Cyclin-dependent kinase E-1	A13	1,179,217	1,180,641	+	1425
	Gh_D09G1794	CDKE-1	Cyclin-dependent kinase E-1	D09	45,602,368	45,603,810	+	1443
	Gh_D13G0113	CDKE-1	Cyclin-dependent kinase E-1	D13	1,148,889	1,150,328	+	1440
	Gh_A05G0178	CDKF-1	Cyclin-dependent kinase F-1	A05	1,861,427	1,863,742	−	2316
	Gh_A07G0040	CDKF-1	Cyclin-dependent kinase F-1	A07	573,679	576,822	+	3144
	Gh_D05G0242	CDKF-1	Cyclin-dependent kinase F-1	D05	2,194,647	2,196,286	−	1640
	Gh_D07G0069	CDKF-1	Cyclin-dependent kinase F-1	D07	683,455	686,592	−	3138
	Gh_A03G1965	CDKF-4	Cyclin-dependent kinase F-4	A03	164,071	171,343	+	7273
	Gh_A08G1357	CDKF-4	Cyclin-dependent kinase F-4	A08	87,774,325	87,778,918	+	4594
	Gh_A12G1847	CDKF-4	Cyclin-dependent kinase F-4	A12	81,080,441	81,083,558	+	3118
	Gh_D03G1838	CDKF-4	Cyclin-dependent kinase F-4	D03	28,784	31,900	−	3117
	Gh_D08G1653	CDKF-4	Cyclin-dependent kinase F-4	D08	52,031,672	52,034,687	+	3016
	Gh_D12G2017	CDKF-4	Cyclin-dependent kinase F-4	D12	53,176,398	53,179,530	+	3133
	Gh_A03G1115	CDKG-2	Cyclin-dependent kinase G-2	A03	81,001,450	81,004,726	−	3277
	Gh_A04G1202	CDKG-2	Cyclin-dependent kinase G-2	A04	62,205,596	62,208,956	+	3361
	Gh_A07G0469	CDKG-2	Cyclin-dependent kinase G-2	A07	6,070,719	6,072,909	−	2191
	Gh_A12G1705	CDKG-2	Cyclin-dependent kinase G-2	A12	78,774,164	78,776,378	+	2215
	Gh_D02G1543	CDKG-2	Cyclin-dependent kinase G-2	D02	53,453,957	53,457,227	−	3271
	Gh_D04G1812	CDKG-2	Cyclin-dependent kinase G-2	D04	50,433,225	50,436,586	+	3362
	Gh_D07G0534	CDKG-2	Cyclin-dependent kinase G-2	D07	6,034,826	6,036,921	−	2096
	Gh_D12G1867	CDKG-2	Cyclin-dependent kinase G-2	D12	51,280,460	51,282,677	+	2218
*G. raimondii (DD)*	Gorai.012G047600	CDKB1-2	Cyclin-dependent kinase B1-2	Chr12	5,984,971	5,987,585	+	2615
	Gorai.004G175700	CDKC-1	Cyclin-dependent kinase C-1	Chr04	47,893,134	47,898,215	−	5082
	Gorai.006G057400	CDKD-1	Cyclin-dependent kinase D-1	Chr06	21,087,092	21,092,990	+	5899
	Gorai.006G193300	CDKD-1	Cyclin-dependent kinase D-1	Chr06	45,020,096	45,028,617	+	8522
	Gorai.006G206400	CDKE-1	Cyclin-dependent kinase E-1	Chr06	46,208,009	46,210,865	+	2857
	Gorai.013G013100	CDKE-1	Cyclin-dependent kinase E-1	Chr13	885,517	888,039	+	2523
	Gorai.001G006600	CDKF-1	Cyclin-dependent kinase F-1	Chr01	593,026	596,558	−	3533
	Gorai.009G026100	CDKF-1	Cyclin-dependent kinase F-1	Chr09	1,996,952	1,999,805	−	2,854
	Gorai.003G187100	CDKF-4	Cyclin-dependent kinase F-4	Chr03	45,739,175	45,742,291	+	3117
	Gorai.004G178800	CDKF-4	Cyclin-dependent kinase F-4	Chr04	48,759,184	48,764,623	+	5440
	Gorai.008G220700	CDKF-4	Cyclin-dependent kinase F-4	Chr08	50,729,473	50,732,623	+	3151
	Gorai.001G060800	CDKG-2	Cyclin-dependent kinase G-2	Chr01	6,021,347	6,025,975	−	4629
	Gorai.005G170300	CDKG-2	Cyclin-dependent kinase G-2	Chr05	49,845,864	49,852,364	−	6501
	Gorai.008G205000	CDKG-2	Cyclin-dependent kinase G-2	Chr08	49,026,157	49,029,075	+	2919
	Gorai.012G174900	CDKG-2	Cyclin-dependent kinase G-2	Chr12	34,446,565	34,450,903	+	4339
*G. arboretum (AA)*	Cotton_A_13039	CDKD-1	Cyclin-dependent kinase D-1	Chr11	62,348,669	62,353,166	+	4498
	Cotton_A_13038	CDKD-1	Cyclin-dependent kinase D-1	Chr11	62,353,469	62,360,835	+	7367
	Cotton_A_11170	CDKF-1	Cyclin-dependent kinase F-1	Chr11	56,386,909	56,389,193	+	2285
	Cotton_A_07964	CDKF-4	Cyclin-dependent kinase F-4	Chr06	49,941,928	49,944,975	+	3048
	Cotton_A_25379	CDKF-4	Cyclin-dependent kinase F-4	Chr10	112,331,167	112,334,076	−	2910
	Cotton_A_14275	CDKF-4	Cyclin-dependent kinase F-4	Chr07	19,382,779	19,385,783	+	3005
	Cotton_A_01035	CDKE-1	Cyclin-dependent kinase E-1	Chr13	75,011,456	75,013,871	−	2416
	Cotton_A_10347	CDKF-1	Cyclin-dependent kinase F-1	Chr01	768,252	771,750	−	3499
	Cotton_A_13019	CDKC-2	Cyclin-dependent kinase C-2	Chr03	85,375,450	85,379,809	−	4360
	Cotton_A_19907	CDKG-2	Cyclin-dependent kinase G-2	Chr01	90,376,726	90,378,745	+	2020
	Cotton_A_08058	CDKG-2	Cyclin-dependent kinase G-2	Chr06	114,700,563	114,702,777	+	2215
	Cotton_A_14138	CDKG-2	Cyclin-dependent kinase G-2	Chr12	122,143,663	122,147,697	+	4035

## Supplementary Materials

Supplementary materials are available at http://www.mdpi.com/1422-0067/19/9/2625/s1.

## Abbreviations

CDK	Cyclin Dependent Kinase
SOD	Superoxide Dismutase
ABRE 4	Abscisic acid (ABA)-Responsive Element (ABRE)-Binding Transcription Factors 4
LEA	Late Embryogenesis Abundant
RD29A	Responsive Drought 29A
POD	Peroxidase
CBL1	Calcineurin B-like protein 1
CAT	Catalase

## References

[1] Queralt E., Uhlmann F. (2008). Cdk-counteracting phosphatases unlock mitotic exit. Curr. Opin. Cell Biol..

[2] Malumbres M. (2014). Cyclin-dependent kinases. Genome Biol..

[3] Loyer P., Trembley J.H., Katona R., Kidd V.J., Lahti J.M. (2005). Role of CDK/cyclin complexes in transcription and RNA splicing. Cell. Signal..

[4] Morgan D.O. (1997). CYCLIN-DEPENDENT KINASES: Engines, Clocks, and Microprocessors. Annu. Rev. Cell Dev. Biol..

[5] Campo S., Baldrich P., Messeguer J., Lalanne E., Coca M., San Segundo B. (2014). Overexpression of a Calcium-Dependent Protein Kinase Confers Salt and Drought Tolerance in Rice by Preventing Membrane Lipid Peroxidation. Plant Physiol..

[6] Bartels D., Sunkar R. (2005). Drought and salt tolerance in plants. CRC Crit. Rev. Plant Sci..

[7] Zhou M., Sun G., Sun Z., Tang Y., Wu Y. (2014). Cotton proteomics for deciphering the mechanism of environment stress response and fiber development. J. Proteom..

[8] Singh R., Pandey N., Kumar A., Shirke P.A. (2016). Physiological performance and differential expression profiling of genes associated with drought tolerance in root tissue of four contrasting varieties of two Gossypium species. Protoplasma.

[9] Kuppu S., Mishra N., Hu R., Sun L., Zhu X., Shen G., Blumwald E., Payton P., Zhang H. (2013). Water-Deficit Inducible Expression of a Cytokinin Biosynthetic Gene IPT Improves Drought Tolerance in Cotton. PLoS ONE.

[10] Mir R.R., Zaman-Allah M., Sreenivasulu N., Trethowan R., Varshney R.K. (2012). Integrated genomics, physiology and breeding approaches for improving drought tolerance in crops. Theor. Appl. Genet..

[11] Weston D.J., Gunter L.E., Rogers A., Wullschleger S.D. (2008). Connecting Genes, Coexpression Modules, and Molecular Signatures to Environmental Stress Phenotypes in Plants. BMC Syst. Biol..

[12] Sofia A., de Almeida A.M., da Silva A.B., da Silva J.M., Paula A., Santos D., Fevereiro P., Sousa Araujo S. (2013). de Abiotic Stress Responses in Plants: Unraveling the Complexity of Genes and Networks to Survive. Abiotic Stress Plant Responses Appl. Agric..

[13] Sakai S., Harada Y., Biology E., Sakai S., Harada Y. (2001). Sink-limitation and the size-number trade-off of organs: Production of organs using a fixed amount of reserves. Evolution.

[14] Tank J.G., Thaker V.S. (2011). Cyclin dependent kinases and their role in regulation of plant cell cycle. Biol. Plant..

[15] Ma X., Qiao Z., Chen D., Yang W., Zhou R., Zhang W., Wang M. (2015). CYCLIN-DEPENDENT KINASE G2 regulates salinity stress response and salt mediated flowering in *Arabidopsis thaliana*. Plant Mol. Biol..

[16] De Veylder L., Joubès J., Inzé D. (2003). Plant cell cycle transitions. Curr. Opin. Plant Biol..

[17] Hayles J., Beach D., Durkacz B., Nurse P. (1986). The fission yeast cell cycle control gene cdc2: Isolation of a sequence suc1 that suppresses cdc2 mutant function. Mol. Gen. Genet..

[18] Hadwiger J.A., Wittenberg C., Mendenhall M.D., Reed S.I. (1989). The Saccharomyces cerevisiae CKS1 gene, a homolog of the S. pombe suc1 gene, encodes a subunit of the cdc28 protein kinase complex. Mol. Cell. Biol..

[19] De Veylder L., Segers G., Glab N., Casteels P., Van Montagu M., Inzé D. (1997). The arabidopsis Cks1At protein binds the cyclin-dependent kinases Cdc2aAt and Cdc2bAt. FEBS Lett..

[20] De Veylder L., Beemster G.T.S., Beeckman T., Inzé D. (2001). CKS1At overexpression in *Arabidopsis thaliana* inhibits growth by reducing meristem size and inhibiting cell-cycle progression. Plant J..

[21] Sacks M.M., Silk W.K., Burman P. (1997). Effect of Water Stress on Cortical Cell Division Rates within the Apical Meristem of Primary Roots of Maize. Plant Physiol..

[22] Schuppler U., He P., John P., Munns R. (1998). Effect of water stress on cell division and cell-division-cycle 2-like cell-cycle kinase activity in wheat leaves. Plant Physiol..

[23] Zhou X.F., Jin Y.H., Yoo C.Y., Lin X.-L., Kim W.-Y., Yun D.-J., Bressan R.A., Hasegawa P.M., Jin J.B. (2013). CYCLIN H;1 Regulates Drought Stress Responses and Blue Light-Induced Stomatal Opening by Inhibiting Reactive Oxygen Species Accumulation in Arabidopsis. Plant Physiol..

[24] Zhao L., Li Y., Xie Q., Wu Y. (2017). Loss of CDKC;2 increases both cell division and drought tolerance in *Arabidopsis thaliana*. Plant J..

[25] Finn R.D., Clements J., Eddy S.R. (2011). HMMER web server: Interactive sequence similarity searching. Nucleic Acids Res..

[26] Magwanga R.O., Lu P., Kirungu J.N., Dong Q., Hu Y., Zhou Z., Cai X., Wang X., Hou Y., Wang K. (2018). Cotton late embryogenesis abundant (*lea2*) genes promote root growth and confers drought stress tolerance in transgenic *Arabidopsis Thaliana*. G3 (Bethesda).

[27] Wang G. (2004). Genome-Wide Analysis of the Cyclin Family in Arabidopsis and Comparative Phylogenetic Analysis of Plant Cyclin-Like Proteins. Plant Physiol..

[28] Fuxreiter M., Simon I., Friedrich P., Tompa P. (2004). Preformed structural elements feature in partner recognition by intrinsically unstructured proteins. J. Mol. Biol..

[29] Hu R., Qi G., Kong Y., Kong D., Gao Q., Zhou G. (2010). Comprehensive analysis of NAC domain transcription factor gene family in *Populus trichocarpa*. BMC Plant Biol..

[30] Gupta S., Stamatoyannopoulos J.A., Bailey T.L., Noble W.S. (2007). Quantifying similarity between motifs. Genome Biol..

[31] Tao X., Gu Y.H., Wang H.Y., Zheng W., Li X., Zhao C.W., Zhang Y.Z. (2012). Digital gene expression analysis based on integrated de novo transcriptome assembly of sweet potato [*Ipomoea batatas (L.) Lam*]. PLoS ONE.

[32] Lee M.-K., Zhang Y., Zhang M., Goebel M., Kim H.J., Triplett B.A., Stelly D.M., Zhang H.-B. (2013). Construction of a plant-transformation-competent BIBAC library and genome sequence analysis of polyploid Upland cotton (*Gossypium hirsutum L.*). BMC Genom..

[33] Salama S.R., Hendricks K.B., Thorner J. (1994). G1 cyclin degradation: The PEST motif of yeast Cln2 is necessary, but not sufficient, for rapid protein turnover. Mol. Cell. Biol..

[34] Bies-Ethève N., Gaubier-Comella P., Debures A., Lasserre E., Jobet E., Raynal M., Cooke R., Delseny M. (2008). Inventory, evolution and expression profiling diversity of the LEA (late embryogenesis abundant) protein gene family in *Arabidopsis thaliana*. Plant Mol. Biol..

[35] Philippe R., Courtois B., McNally K.L., Mournet P., El-Malki R., Le Paslier M.C., Fabre D., Billot C., Brunel D., Glaszmann J.C. (2010). Structure, allelic diversity and selection of Asr genes, candidate for drought tolerance, in *Oryza sativa* L. and wild relatives. Theor. Appl. Genet..

[36] Xu G., Guo C., Shan H., Kong H. (2012). Divergence of duplicate genes in exon-intron structure. Proc. Natl. Acad. Sci. USA.

[37] Liu Z., Adams K.L. (2007). Expression Partitioning between Genes Duplicated by Polyploidy under Abiotic Stress and during Organ Development. Curr. Biol..

[38] Salih H., Gong W., He S., Sun G., Sun J., Du X. (2016). Genome-wide characterization and expression analysis of MYB transcription factors in *Gossypium hirsutum*. BMC Genet..

[39] Ng P.C., Henikoff S. (2003). SIFT: Predicting amino acid changes that affect protein function. Nucleic Acids Res..

[40] Cao J., Li X. (2014). Identification and phylogenetic analysis of late embryogenesis abundant proteins family in tomato (*Solanum lycopersicum*). Planta.

[41] Hundertmark M., Hincha D.K. (2008). LEA (Late Embryogenesis Abundant) proteins and their encoding genes in *Arabidopsis thaliana*. BMC Genom..

[42] Du D., Zhang Q., Cheng T., Pan H., Yang W., Sun L. (2013). Genome-wide identification and analysis of late embryogenesis abundant (LEA) genes in Prunus mume. Mol. Biol. Rep..

[43] Yamaguchi-Shinozaki K., Shinozaki K. (2005). Organization of cis-acting regulatory elements in osmotic- and cold-stress-responsive promoters. Trends Plant Sci..

[44] Li C., Ng C.K.Y., Fan L.M. (2014). MYB transcription factors, active players in abiotic stress signaling. Environ. Exp. Bot..

[45] De Azevedo Neto A.D., Prisco J.T., Enéas-Filho J., Abreu C.E.B.D., Gomes-Filho E. (2006). Effect of salt stress on antioxidative enzymes and lipid peroxidation in leaves and roots of salt-tolerant and salt-sensitive maize genotypes. Environ. Exp. Bot..

[46] Tamirisa S., Vudem D.R., Khareedu V.R. (2017). A Cyclin Dependent Kinase Regulatory Subunit (CKS) Gene of Pigeonpea Imparts Abiotic Stress Tolerance and Regulates Plant Growth and Development in Arabidopsis. Front. Plant Sci..

[47] Zhang L., Tian L.-H., Zhao J.-F., Song Y., Zhang C.-J., Guo Y. (2009). Identification of an apoplastic protein involved in the initial phase of salt stress response in rice root by two-dimensional electrophoresis. Plant Physiol..

[48] Zörb C., Schmitt S., Neeb A., Karl S., Linder M., Schubert S. (2004). The biochemical reaction of maize (*Zea mays L.*) to salt stress is characterized by a mitigation of symptoms and not by a specific adaptation. Plant Sci..

[49] Xu C., Sibicky T., Huang B. (2010). Protein profile analysis of salt-responsive proteins in leaves and roots in two cultivars of creeping bentgrass differing in salinity tolerance. Plant Cell Rep..

[50] Abdollahi M.R., Corral-Martínez P., Mousavi A., Salmanian A.H., Moieni A., Seguí-Simarro J.M. (2009). An efficient method for transformation of pre-androgenic, isolated Brassica napus microspores involving microprojectile bombardment and Agrobacterium-mediated transformation. Acta Physiol. Plant..

[51] Kurusu T., Nishikawa D., Yamazaki Y., Gotoh M., Nakano M., Hamada H., Yamanaka T., Iida K., Nakagawa Y., Saji H. (2012). Plasma membrane protein OsMCA1 is involved in regulation of hypo-osmotic shock-induced Ca^2+^ influx and modulates generation of reactive oxygen species in cultured rice cells. BMC Plant Biol..

[52] Ganança J.F.T., Freitas J.G.R., Nóbrega H.G.M., Rodrigues V., Antunes G., Gouveia C.S.S., Rodrigues M., Chaïr H., De Carvalho M.Â.A.P., Lebot V. (2018). Screening for drought tolerance in thirty three taro cultivars. Not. Bot. Horti Agrobot. Cluj-Napoca.

[53] Pandey H.C., Baig M.J., Chandra A., Bhatt R.K. (2010). Drought stress induced changes in lipid peroxidation and antioxidant system in genus Avena. J. Environ. Biol..

[54] Lu P., Magwanga R.O., Lu H., Kirungu J.N., Wei Y., Dong Q., Wang X., Cai X., Zhou Z., Wang K. (2018). A novel G-protein-coupled receptors gene from upland cotton enhances salt stress tolerance in transgenic *Arabidopsis*. Genes (Basel).

[55] Magwanga R.O., Lu P., Kirungu J.N., Lu H., Wang X., Cai X., Zhou Z., Zhang Z., Salih H., Wang K. (2018). Characterization of the late embryogenesis abundant (LEA) proteins family and their role in drought stress tolerance in upland cotton. BMC Genet..

[56] Rong J., Feltus F.A., Liu L., Lin L., Paterson A.H. (2010). Gene copy number evolution during tetraploid cotton radiation. Heredity (Edinb).

[57] Guo J., Song J., Wang F., Zhang X.S. (2007). Genome-wide identification and expression analysis of rice cell cycle genes. Plant Mol. Biol..

[58] Zou C., Lehti-Shiu M.D., Thomashow M., Shiu S.-H. (2009). Evolution of stress-regulated gene expression in duplicate genes of *Arabidopsis thaliana*. PLoS Genet..

[59] Flagel L.E., Wendel J.E. (2009). Gene duplication and evolutionaary novelty in plants. New Phytol..

[60] Krasensky J., Jonak C. (2012). Drought, salt, and temperature stress-induced metabolic rearrangements and regulatory networks. J. Exp. Bot..

[61] Blanc G., Hokamp K., Wolfe K.H. (2003). A recent polyploidy superimposed on older large-scale duplications in the *Arabidopsis* genome. Genome Res..

[62] Vandepoele K., Raes J., De Veylder L., Rouze P., Rombauts S., Inze D. (2002). Genome-wide analysis of core cell cycle genes in Arabidopsis. Plant Cell.

[63] Dossa K., Diouf D., Cissé N. (2016). Genome-Wide Investigation of Hsf Genes in Sesame Reveals Their Segmental Duplication Expansion and Their Active Role in Drought Stress Response. Front. Plant Sci..

[64] Grant D., Cregan P., Shoemaker R.C. (2000). Genome organization in dicots: Genome duplication in Arabidopsis and synteny between soybean and *Arabidopsis*. Proc. Natl. Acad. Sci. USA.

[65] Zhang T., Wang X., Lu Y., Cai X., Ye Z., Zhang J. (2013). Genome-wide analysis of the cyclin gene family in tomato. Int. J. Mol. Sci..

[66] Lan T., Gao J., Zeng Q.Y. (2013). Genome-wide analysis of the LEA (late embryogenesis abundant) protein gene family in *Populus trichocarpa*. Tree Genet. Genomes.

[67] Wahl M.C., Will C.L., Lührmann R. (2009). The Spliceosome: Design Principles of a Dynamic RNP Machine. Cell.

[68] Guimarães-Dias F., Neves-Borges A.C., Viana A.A.B., Mesquita R.O., Romano E., de Fátima Grossi-de-Sá M., Nepomuceno A.L., Loureiro M.E., Alves-Ferreira M. (2012). Expression analysis in response to drought stress in soybean: Shedding light on the regulation of metabolic pathway genes. Genet. Mol. Biol..

[69] Hajiboland R., Parvaiz A. (2014). Chapter 1—Reactive Oxygen Species and Photosynthesis. Oxidative Damage to Plants.

[70] Jo B.H., Van Lerberghe L.M., Motsegood K.M., Beebe D.J., Fuchs T.A., Abed U., Goosmann C., Hurwitz R., Schulze I., Wahn V. (2010). Novel cell death program leads to neutrophil extracellular traps. J. Cell Biol..

[71] Msanne J., Lin J., Stone J.M., Awada T. (2011). Characterization of abiotic stress-responsive *Arabidopsis thaliana* RD29A and RD29B genes and evaluation of transgenes. Planta.

[72] Horton P., Park K.J., Obayashi T., Fujita N., Harada H., Adams-Collier C.J., Nakai K. (2007). WoLF PSORT: Protein localization predictor. Nucleic Acids Res..

[73] Voorrips R.E. (2002). MapChart: Software for the graphical presentation of linkage maps and QTLs. J. Hered..

[74] Du D., Hao R., Cheng T., Pan H., Yang W., Wang J., Zhang Q. (2013). Genome-Wide Analysis of the AP2/ERF Gene Family in Prunus mume. Plant Mol. Biol. Rep..

[75] Tamura K., Stecher G., Peterson D., Filipski A., Kumar S. (2013). MEGA6: Molecular evolutionary genetics analysis version 6.0. Mol. Biol. Evol..

[76] R Development Core Team (2008). R Software.

[77] Hoagland D.R., Arnon D.I. (1950). The Water-Culture Method for Growing Plants Without Soil.

[78] Parent B., Suard B., Serraj R., Tardieu F. (2010). Rice leaf growth and water potential are resilient to evaporative demand and soil water deficit once the effects of root system are neutralized. Plant Cell Environ..

[79] Clough S.J., Bent A.F. (1998). Floral dip: A simplified method for Agrobacterium-mediated transformation of *Arabidopsis thaliana*. Plant J..

[80] Benoit R.M., Ostermeier C., Geiser M., Li J.S.Z., Widmer H., Auer M. (2016). Seamless insert-plasmid assembly at high efficiency and low cost. PLoS ONE.

[81] Sun W., Cao Z., Li Y., Zhao Y., Zhang H. (2007). A simple and effective method for protein subcellular localization using Agrobacterium-mediated transformation of onion epidermal cells. Biologia (Bratisl).

[82] Guo Y.Y., Yu H.Y., Yang M.M., Kong D.S., Zhang Y.J. (2018). Effect of Drought Stress on Lipid Peroxidation, Osmotic Adjustment and Antioxidant Enzyme Activity of Leaves and Roots of Lycium ruthenicum Murr. Seedling. Russ. J. Plant Physiol..

[83] de BritoI G.G., Sofiatti V., de Andrade Lima M.M., de Carvalho L.P., da Silva Filho J.L. (2011). Physiological traits for drought phenotyping in cotton. Acta Sci. Agron..

