# Supplementary material for: Whole Genome Analysis of Cyclin Dependent Kinase (CDK) Gene Family in Cotton and Functional Evaluation of the Role of CDKF4 Gene in Drought and Salt Stress Tolerance in Plants
Source: Int J Mol Sci. 2018 Sep 5;19(9):2625. doi: 10.3390/ijms19092625 (PMC6164816; doi:10.3390/ijms19092625)
Supplement: Supplementary file 1 [file ijms-19-02625-s001.zip › Supplementary materials/Supplementary Table 1 Cotton CDK genes and properties of the deduced proteins.docx]

**Supplementary Table 1:** Cotton *CDK* genes and properties of the deduced proteins

| Gene ID | Gene Name | Description | Chro. | Start | End | Strand | Length (bp) | Protein Length (aa) | Molecular Weight (kDa) | Charge | Isoelectric Point | GRAVY | Exon Number | Mean Exon Length (bp) | Mean Intron Length (bp) |
| --- | --- | --- | --- | --- | --- | --- | --- | --- | --- | --- | --- | --- | --- | --- | --- |
| Cotton_A_01035 | CDKE-1 | Cyclin-dependent kinase E-1 | Chr13 | 75,011,456 | 75,013,871 | - | 2,416 | 475 | 53.052 | 18 | 9.636 | -0.462 | 1 | 2,416.00 | No intron |
| Cotton_A_07964 | CDKF-4 | Cyclin-dependent kinase F-4 | Chr06 | 49,941,928 | 49,944,975 | + | 3,048 | 437 | 49.942 | 26.5 | 9.972 | -0.35 | 16 | 81.9 | 115.8 |
| Cotton_A_08058 | CDKG-2 | Cyclin-dependent kinase G-2 | Chr06 | 114,700,563 | 114,702,777 | + | 2,215 | 603 | 68.342 | -8.5 | 5.477 | -0.553 | 5 | 362.4 | 100.8 |
| Cotton_A_10347 | CDKF-1 | Cyclin-dependent kinase F-1 | Chr01 | 768,252 | 771,750 | - | 3,499 | 475 | 53.401 | -29.5 | 4.34 | -0.397 | 2 | 891.5 | 1,716.00 |
| Cotton_A_11170 | CDKF-1 | Cyclin-dependent kinase F-1 | Chr11 | 56,386,909 | 56,389,193 | + | 2,285 | 425 | 47.552 | -14.5 | 4.669 | -0.342 | 6 | 212.5 | 202 |
| Cotton_A_13019 | CDKC-2 | Cyclin-dependent kinase C-2 | Chr03 | 85,375,450 | 85,379,809 | - | 4,360 | 518 | 57.286 | 26.5 | 9.677 | 0 | 12 | 129.5 | 255.1 |
| Cotton_A_13038 | CDKD-1 | Cyclin-dependent kinase D-1 | Chr11 | 62,353,469 | 62,360,835 | + | 7,367 | 410 | 46.421 | 16 | 9.889 | -0.425 | 7 | 175.7 | 1,022.80 |
| Cotton_A_13039 | CDKD-1 | Cyclin-dependent kinase D-1 | Chr11 | 62,348,669 | 62,353,166 | + | 4,498 | 375 | 42.419 | 14.5 | 9.811 | -0.382 | 7 | 161.1 | 561.7 |
| Cotton_A_14138 | CDKG-2 | Cyclin-dependent kinase G-2 | Chr12 | 122,143,663 | 122,147,697 | + | 4,035 | 814 | 91.144 | 0.5 | 6.573 | -0.668 | 8 | 305.6 | 227.1 |
| Cotton_A_14275 | CDKF-4 | Cyclin-dependent kinase F-4 | Chr07 | 19,382,779 | 19,385,783 | + | 3,005 | 456 | 51.975 | 24.5 | 10.009 | -0.468 | 16 | 85.7 | 108.9 |
| Cotton_A_19907 | CDKG-2 | Cyclin-dependent kinase G-2 | Chr01 | 90,376,726 | 90,378,745 | + | 2,020 | 514 | 58.296 | -15 | 4.794 | -0.502 | 5 | 309 | 118.8 |
| Cotton_A_25379 | CDKF-4 | Cyclin-dependent kinase F-4 | Chr10 | 112,331,167 | 112,334,076 | - | 2,910 | 447 | 51.035 | 30.5 | 10.323 | -0.387 | 16 | 84 | 118.5 |
| Gh_A03G1115 | CDKG-2 | Cyclin-dependent kinase G-2 | A03 | 81,001,450 | 81,004,726 | - | 3,277 | 754 | 84.802 | 0 | 6.524 | -0.738 | 6 | 377.5 | 202.4 |
| Gh_A03G1965 | CDKF-4 | Cyclin-dependent kinase F-4 | scaffold | 164,071 | 171,343 | + | 7,273 | 689 | 78.915 | 28.5 | 9.468 | -0.156 | 23 | 90 | 236.5 |
| Gh_A04G1202 | CDKG-2 | Cyclin-dependent kinase G-2 | A04 | 62,205,596 | 62,208,956 | + | 3,361 | 744 | 83.332 | -1 | 6.426 | -0.785 | 6 | 372.5 | 225.2 |
| Gh_A05G0178 | CDKF-1 | Cyclin-dependent kinase F-1 | A05 | 1,861,427 | 1,863,742 | - | 2,316 | 328 | 37.341 | -5 | 5.461 | -0.399 | 3 | 329 | 664.5 |
| Gh_A07G0040 | CDKF-1 | Cyclin-dependent kinase F-1 | A07 | 573,679 | 576,822 | + | 3,144 | 475 | 53.387 | -29.5 | 4.34 | -0.402 | 2 | 714 | 1,716.00 |
| Gh_A07G0469 | CDKG-2 | Cyclin-dependent kinase G-2 | A07 | 6,070,719 | 6,072,909 | - | 2,191 | 571 | 64.659 | -11 | 5.019 | -0.529 | 5 | 343.2 | 118.8 |
| Gh_A08G1333 | CDKC-1 | Cyclin-dependent kinase C-1 | A08 | 86,315,556 | 86,319,915 | - | 4,360 | 516 | 57.022 | 24.5 | 9.568 | -0.791 | 12 | 129.3 | 255.4 |
| Gh_A08G1357 | CDKF-4 | Cyclin-dependent kinase F-4 | A08 | 87,774,325 | 87,778,918 | + | 4,594 | 547 | 62.002 | 30.5 | 10.043 | -0.36 | 18 | 91.3 | 173.5 |
| Gh_A09G0392 | CDKB1-2 | Cyclin-dependent kinase B1-2 | A09 | 25,358,935 | 25,360,412 | + | 1,478 | 120 | 13.817 | -1 | 6.089 | -0.315 | 3 | 121 | 557.5 |
| Gh_A09G0498 | CDKD-1 | Cyclin-dependent kinase D-1 | A09 | 39,565,295 | 39,569,539 | - | 4,245 | 413 | 46.854 | 12.5 | 9.727 | -0.385 | 7 | 177.4 | 500.5 |
| Gh_A09G1581 | CDKD-1 | Cyclin-dependent kinase D-1 | A09 | 69,169,679 | 69,177,142 | + | 7,464 | 410 | 46.32 | 16 | 9.889 | -0.417 | 7 | 176.1 | 1,038.50 |
| Gh_A09G1688 | CDKE-1 | Cyclin-dependent kinase E-1 | A09 | 70,391,997 | 70,393,439 | + | 1,443 | 480 | 53.417 | 18 | 9.646 | -0.458 | 1 | 1,443.00 | No intron |
| Gh_A12G1705 | CDKG-2 | Cyclin-dependent kinase G-2 | A12 | 78,774,164 | 78,776,378 | + | 2,215 | 603 | 68.36 | -8.5 | 5.479 | -0.548 | 5 | 362.4 | 100.8 |
| Gh_A12G1847 | CDKF-4 | Cyclin-dependent kinase F-4 | A12 | 81,080,441 | 81,083,558 | + | 3,118 | 432 | 49.233 | 24.5 | 9.757 | -0.391 | 16 | 81.2 | 121.3 |
| Gh_A13G0098 | CDKE-1 | Cyclin-dependent kinase E-1 | A13 | 1,179,217 | 1,180,641 | + | 1,425 | 474 | 52.938 | 18 | 9.636 | -0.455 | 1 | 1,425.00 | No intron |
| Gh_D02G1543 | CDKG-2 | Cyclin-dependent kinase G-2 | D02 | 53,453,957 | 53,457,227 | - | 3,271 | 754 | 85.049 | 1 | 6.607 | -0.779 | 6 | 377.5 | 201.2 |
| Gh_D03G1838 | CDKF-4 | Cyclin-dependent kinase F-4 | scaffold | 28,784 | 31,900 | - | 3,117 | 459 | 52.559 | 29 | 10.242 | -0.332 | 16 | 86.3 | 115.8 |
| Gh_D04G0378 | CDKB1-2 | Cyclin-dependent kinase B1-2 | D04 | 5,886,575 | 5,888,627 | + | 2,053 | 307 | 34.92 | 8.5 | 8.56 | -0.325 | 3 | 308 | 564.5 |
| Gh_D04G1812 | CDKG-2 | Cyclin-dependent kinase G-2 | D04 | 50,433,225 | 50,436,586 | + | 3,362 | 744 | 83.195 | -1.5 | 6.362 | -0.792 | 6 | 372.5 | 225.4 |
| Gh_D05G0242 | CDKF-1 | Cyclin-dependent kinase F-1 | D05 | 2,194,647 | 2,196,286 | - | 1,640 | 210 | 23.517 | -7.5 | 4.637 | -0.467 | 2 | 316.5 | 1,007.00 |
| Gh_D07G0069 | CDKF-1 | Cyclin-dependent kinase F-1 | D07 | 683,455 | 686,592 | - | 3,138 | 472 | 53.043 | -28.5 | 4.359 | -0.399 | 2 | 709.5 | 1,719.00 |
| Gh_D07G0534 | CDKG-2 | Cyclin-dependent kinase G-2 | D07 | 6,034,826 | 6,036,921 | - | 2,096 | 571 | 64.707 | -13 | 4.928 | -0.568 | 5 | 343.2 | 95 |
| Gh_D08G1628 | CDKC-2 | Cyclin-dependent kinase C-2 | D08 | 51,109,124 | 51,113,530 | - | 4,407 | 518 | 57.255 | 26.5 | 9.677 | -0.792 | 12 | 129.8 | 259.1 |
| Gh_D08G1653 | CDKF-4 | Cyclin-dependent kinase F-4 | D08 | 52,031,672 | 52,034,687 | + | 3,016 | 460 | 52.475 | 24.5 | 9.961 | -0.481 | 16 | 86.4 | 108.9 |
| Gh_D09G0505 | CDKD-1 | Cyclin-dependent kinase D-1 | D09 | 24,483,522 | 24,487,751 | - | 4,230 | 413 | 46.868 | 14.5 | 9.874 | -0.412 | 7 | 177.4 | 498 |
| Gh_D09G1668 | CDKD-1 | Cyclin-dependent kinase D-1 | D09 | 44,329,951 | 44,337,731 | + | 7,781 | 413 | 46.443 | 15 | 9.904 | -0.34 | 7 | 177.4 | 1,089.80 |
| Gh_D09G1794 | CDKE-1 | Cyclin-dependent kinase E-1 | D09 | 45,602,368 | 45,603,810 | + | 1,443 | 480 | 53.386 | 18 | 9.646 | -0.448 | 1 | 1,443.00 | No intron |
| Gh_D12G1867 | CDKG-2 | Cyclin-dependent kinase G-2 | D12 | 51,280,460 | 51,282,677 | + | 2,218 | 574 | 64.962 | -11 | 5.148 | -0.6 | 6 | 287.5 | 98.6 |
| Gh_D12G2017 | CDKF-4 | Cyclin-dependent kinase F-4 | D12 | 53,176,398 | 53,179,530 | + | 3,133 | 432 | 49.284 | 22.5 | 9.678 | -0.392 | 16 | 81.2 | 122.3 |
| Gh_D13G0113 | CDKE-1 | Cyclin-dependent kinase E-1 | D13 | 1,148,889 | 1,150,328 | + | 1,440 | 479 | 53.416 | 18.5 | 9.636 | -0.437 | 1 | 1,440.00 | No intron |
| Gorai.001G006600 | CDKF-1 | Cyclin-dependent kinase F-1 | Chr01 | 593,026 | 596,558 | - | 3,533 | 475 | 53.365 | -29.5 | 4.34 | -0.403 | 2 | 907 | 1,719.00 |
| Gorai.001G060800 | CDKG-2 | Cyclin-dependent kinase G-2 | Chr01 | 6,021,347 | 6,025,975 | - | 4,629 | 571 | 64.662 | -13 | 4.93 | -0.548 | 7 | 585.6 | 88.3 |
| Gorai.003G187100 | CDKF-4 | Cyclin-dependent kinase F-4 | Chr03 | 45,739,175 | 45,742,291 | + | 3,117 | 425 | 48.674 | 27.5 | 10.233 | -0.436 | 15 | 85.2 | 131.4 |
| Gorai.004G175700 | CDKC-1 | Cyclin-dependent kinase C-1 | Chr04 | 47,893,134 | 47,898,215 | - | 5,082 | 516 | 57.053 | 24.5 | 9.568 | -0.795 | 12 | 186.5 | 258.5 |
| Gorai.004G178800 | CDKF-4 | Cyclin-dependent kinase F-4 | Chr04 | 48,759,184 | 48,764,623 | + | 5,440 | 460 | 52.472 | 24.5 | 9.961 | -0.476 | 19 | 162.4 | 130.8 |
| Gorai.005G170300 | CDKG-2 | Cyclin-dependent kinase G-2 | Chr05 | 49,845,864 | 49,852,364 | - | 6,501 | 754 | 85.043 | 3.5 | 6.838 | -0.776 | 7 | 626 | 353.2 |
| Gorai.006G057400 | CDKD-1 | Cyclin-dependent kinase D-1 | Chr06 | 21,087,092 | 21,092,990 | + | 5,899 | 413 | 46.914 | 13.5 | 9.808 | -0.423 | 8 | 353.4 | 438.9 |
| Gorai.006G193300 | CDKD-1 | Cyclin-dependent kinase D-1 | Chr06 | 45,020,096 | 45,028,617 | + | 8,522 | 410 | 46.367 | 15 | 9.815 | -0.412 | 8 | 268.1 | 911 |
| Gorai.006G206400 | CDKE-1 | Cyclin-dependent kinase E-1 | Chr06 | 46,208,009 | 46,210,865 | + | 2,857 | 480 | 53.404 | 18 | 9.646 | -0.452 | 3 | 768 | 276.5 |
| Gorai.008G205000 | CDKG-2 | Cyclin-dependent kinase G-2 | Chr08 | 49,026,157 | 49,029,075 | + | 2,919 | 635 | 71.812 | -7 | 5.611 | -0.53 | 7 | 272.6 | 168.5 |
| Gorai.008G220700 | CDKF-4 | Cyclin-dependent kinase F-4 | Chr08 | 50,729,473 | 50,732,623 | + | 3,151 | 436 | 49.666 | 25.5 | 9.906 | -0.392 | 16 | 81.9 | 122.7 |
| Gorai.009G026100 | CDKF-1 | Cyclin-dependent kinase F-1 | Chr09 | 1,996,952 | 1,999,805 | - | 2,854 | 344 | 39.042 | -13.5 | 4.687 | -0.443 | 2 | 933.5 | 987 |
| Gorai.012G047600 | CDKB1-2 | Cyclin-dependent kinase B1-2 | Chr12 | 5,984,971 | 5,987,585 | + | 2,615 | 307 | 34.92 | 8.5 | 8.56 | -0.325 | 3 | 494.3 | 566 |
| Gorai.012G174900 | CDKG-2 | Cyclin-dependent kinase G-2 | Chr12 | 34,446,565 | 34,450,903 | + | 4,339 | 744 | 83.245 | -0.5 | 6.479 | -0.776 | 7 | 448.7 | 199.7 |
| Gorai.013G013100 | CDKE-1 | Cyclin-dependent kinase E-1 | Chr13 | 885,517 | 888,039 | + | 2,523 | 479 | 53.392 | 18.5 | 9.636 | -0.447 | 3 | 688.7 | 228.5 |

CDK-cyclin- dependent kinase; B,C,D,F and F refers to the classes of CDKs, the numbers -1 , -2… refers to the sub type in each class
