# Supplementary material for: Whole Genome Analysis of Cyclin Dependent Kinase (CDK) Gene Family in Cotton and Functional Evaluation of the Role of CDKF4 Gene in Drought and Salt Stress Tolerance in Plants
Source: Int J Mol Sci. 2018 Sep 5;19(9):2625. doi: 10.3390/ijms19092625 (PMC6164816; doi:10.3390/ijms19092625)
Supplement: Supplementary file 1 [file ijms-19-02625-s001.zip › Supplementary materials/Supplementary Table 2 Sub cellular localization of the cotton CDK genes, by the use of WoLF PSORT, Target P and Pprowler online too.docx]

**Supplementary Table 2:** Sub cellular localization of the cotton *CDK* genes, by the use of WoLF PSORT, Target P and Pprowler online tool

| Gene ID | Gene  name | WoLF PSORT | TargetP 1.1 prediction | | | | | | | Pprowler | | | |
| --- | --- | --- | --- | --- | --- | --- | --- | --- | --- | --- | --- | --- | --- |
|  |  |  | Len | cTP | mTP | SP | other | Loc | RC | SP | mTP | cTP | OTHER |
| Gh_A03G1115 | CDKG-2 | nucl | 754 | 0.122 | 0.12 | 0.012 | 0.905 | _ | 2 | 0.01 | 0.08 | 0 | 0.91 |
| Gh_A03G1965 | CDKF-4 | plas | 689 | 0.05 | 0.127 | 0.036 | 0.926 | _ | 2 | 0.01 | 0.06 | 0 | 0.93 |
| Gh_A04G1202 | CDKG-2 | nucl | 744 | 0.154 | 0.253 | 0.005 | 0.533 | _ | 4 | 0.02 | 0.14 | 0.01 | 0.83 |
| Gh_A05G0178 | CDKF-1 | cyto | 328 | 0.123 | 0.256 | 0.025 | 0.729 | _ | 3 | 0.02 | 0.1 | 0 | 0.89 |
| Gh_A07G0040 | CDKF-1 | chlo | 475 | 0.101 | 0.262 | 0.039 | 0.662 | _ | 3 | 0.02 | 0.13 | 0 | 0.85 |
| Gh_A07G0469 | CDKG-2 | nucl | 571 | 0.045 | 0.318 | 0.012 | 0.848 | _ | 3 | 0.01 | 0.07 | 0 | 0.91 |
| Gh_A08G1333 | CDKC-1 | nucl | 516 | 0.141 | 0.168 | 0.1 | 0.739 | _ | 3 | 0.02 | 0.08 | 0 | 0.9 |
| Gh_A08G1357 | CDKF-4 | cyto | 547 | 0.024 | 0.125 | 0.13 | 0.75 | _ | 2 | 0.53 | 0.23 | 0.07 | 0.17 |
| Gh_A09G0392 | CDKB1-2 | nucl | 120 | 0.058 | 0.105 | 0.415 | 0.373 | S | 5 | 0.1 | 0.13 | 0.01 | 0.76 |
| Gh_A09G0498 | CDKD-1 | cyto | 413 | 0.034 | 0.171 | 0.08 | 0.897 | _ | 2 | 0.01 | 0.05 | 0 | 0.94 |
| Gh_A09G1581 | CDKD-1 | cyto | 410 | 0.036 | 0.163 | 0.077 | 0.92 | _ | 2 | 0.01 | 0.05 | 0 | 0.94 |
| Gh_A09G1688 | CDKE-1 | cyto | 480 | 0.295 | 0.07 | 0.057 | 0.799 | _ | 3 | 0.01 | 0.07 | 0 | 0.92 |
| Gh_A12G1705 | CDKG-2 | nucl | 603 | 0.034 | 0.529 | 0.036 | 0.724 | _ | 5 | 0.01 | 0.09 | 0 | 0.89 |
| Gh_A12G1847 | CDKF-4 | cyto | 432 | 0.032 | 0.229 | 0.024 | 0.9 | _ | 2 | 0.01 | 0.09 | 0 | 0.89 |
| Gh_A13G0098 | CDKE-1 | cyto | 474 | 0.285 | 0.075 | 0.047 | 0.822 | _ | 3 | 0.01 | 0.06 | 0 | 0.92 |
| Gh_D02G1543 | CDKG-2 | nucl | 754 | 0.121 | 0.11 | 0.009 | 0.904 | _ | 2 | 0.01 | 0.08 | 0 | 0.9 |
| Gh_D03G1838 | CDKF-4 | cyto | 459 | 0.048 | 0.127 | 0.037 | 0.927 | _ | 1 | 0.01 | 0.06 | 0 | 0.93 |
| Gh_D04G0378 | CDKB1-2 | cyto | 307 | 0.152 | 0.111 | 0.054 | 0.906 | _ | 2 | 0.01 | 0.05 | 0 | 0.94 |
| Gh_D04G1812 | CDKG-2 | nucl | 744 | 0.197 | 0.2 | 0.004 | 0.522 | _ | 4 | 0.02 | 0.14 | 0.01 | 0.83 |
| Gh_D05G0242 | CDKF-1 | chlo | 210 | 0.069 | 0.336 | 0.079 | 0.681 | _ | 4 | 0.03 | 0.25 | 0 | 0.72 |
| Gh_D07G0069 | CDKF-1 | chlo | 472 | 0.101 | 0.278 | 0.042 | 0.656 | _ | 4 | 0.02 | 0.13 | 0 | 0.85 |
| Gh_D07G0534 | CDKG-2 | nucl | 571 | 0.045 | 0.334 | 0.02 | 0.857 | _ | 3 | 0.01 | 0.06 | 0 | 0.92 |
| Gh_D08G1628 | CDKC-2 | nucl | 518 | 0.144 | 0.178 | 0.101 | 0.733 | _ | 3 | 0.02 | 0.08 | 0 | 0.9 |
| Gh_D08G1653 | CDKF-4 | cyto | 460 | 0.047 | 0.148 | 0.032 | 0.931 | _ | 2 | 0.01 | 0.06 | 0 | 0.93 |
| Gh_D09G0505 | CDKD-1 | cyto | 413 | 0.04 | 0.176 | 0.073 | 0.892 | _ | 2 | 0.01 | 0.05 | 0 | 0.94 |
| Gh_D09G1668 | CDKD-1 | cyto | 413 | 0.034 | 0.168 | 0.089 | 0.918 | _ | 2 | 0.01 | 0.05 | 0 | 0.94 |
| Gh_D09G1794 | CDKE-1 | cyto | 480 | 0.284 | 0.071 | 0.056 | 0.81 | _ | 3 | 0.01 | 0.07 | 0 | 0.91 |
| Gh_D12G1867 | CDKG-2 | nucl | 574 | 0.032 | 0.519 | 0.031 | 0.739 | _ | 4 | 0.01 | 0.09 | 0 | 0.89 |
| Gh_D12G2017 | CDKF-4 | cyto | 432 | 0.031 | 0.229 | 0.025 | 0.907 | _ | 2 | 0.01 | 0.09 | 0 | 0.89 |
| Gh_D13G0113 | CDKE-1 | cyto | 479 | 0.248 | 0.093 | 0.043 | 0.824 | _ | 3 | 0.01 | 0.06 | 0 | 0.92 |
| Cotton_A_01035 | CDKE-1 | cyto | 475 | 0.285 | 0.075 | 0.047 | 0.822 | _ | 3 | 0.01 | 0.06 | 0 | 0.92 |
| Cotton_A_07964 | CDKF-4 | cyto | 437 | 0.024 | 0.322 | 0.03 | 0.86 | _ | 3 | 0.02 | 0.22 | 0 | 0.76 |
| Cotton_A_08058 | CDKG-2 | nucl | 603 | 0.035 | 0.552 | 0.034 | 0.7 | _ | 5 | 0.01 | 0.09 | 0 | 0.89 |
| Cotton_A_10347 | CDKF-1 | chlo | 475 | 0.101 | 0.255 | 0.039 | 0.672 | _ | 3 | 0.02 | 0.13 | 0 | 0.85 |
| Cotton_A_11170 | CDKF-1 | cyto | 425 | 0.123 | 0.256 | 0.025 | 0.729 | _ | 3 | 0.02 | 0.1 | 0 | 0.89 |
| Cotton_A_13019 | CDKC-2 | nucl | 518 | 0.144 | 0.178 | 0.101 | 0.733 | _ | 3 | 0.02 | 0.08 | 0 | 0.9 |
| Cotton_A_13038 | CDKD-1 | cyto | 410 | 0.034 | 0.168 | 0.089 | 0.918 | _ | 2 | 0.01 | 0.05 | 0 | 0.94 |
| Cotton_A_13039 | CDKD-1 | cyto | 375 | 0.034 | 0.168 | 0.089 | 0.918 | _ | 2 | 0.01 | 0.05 | 0 | 0.94 |
| Cotton_A_14138 | CDKG-2 | nucl | 814 | 0.147 | 0.233 | 0.004 | 0.56 | _ | 4 | 0.02 | 0.14 | 0.01 | 0.83 |
| Cotton_A_14275 | CDKF-4 | cyto | 456 | 0.047 | 0.148 | 0.032 | 0.931 | _ | 2 | 0.01 | 0.06 | 0 | 0.93 |
| Cotton_A_19907 | CDKG-2 | nucl | 514 | 0.201 | 0.155 | 0.014 | 0.761 | _ | 3 | 0.01 | 0.07 | 0.01 | 0.9 |
| Cotton_A_25379 | CDKF-4 | cyto | 447 | 0.048 | 0.127 | 0.037 | 0.927 | _ | 1 | 0.01 | 0.06 | 0 | 0.93 |
| Gorai.001G006600 | CDKF-1 | chlo | 475 | 0.1 | 0.276 | 0.041 | 0.664 | _ | 4 | 0.02 | 0.13 | 0 | 0.85 |
| Gorai.001G060800 | CDKG-2 | nucl | 571 | 0.045 | 0.333 | 0.02 | 0.859 | _ | 3 | 0.01 | 0.06 | 0 | 0.92 |
| Gorai.003G187100 | CDKF-4 | cyto | 425 | 0.047 | 0.135 | 0.037 | 0.922 | _ | 2 | 0.01 | 0.06 | 0 | 0.93 |
| Gorai.004G175700 | CDKC-1 | nucl | 516 | 0.141 | 0.168 | 0.1 | 0.739 | _ | 3 | 0.02 | 0.08 | 0 | 0.9 |
| Gorai.004G178800 | CDKF-4 | cyto | 460 | 0.047 | 0.148 | 0.032 | 0.931 | _ | 2 | 0.01 | 0.06 | 0 | 0.93 |
| Gorai.005G170300 | CDKG-2 | nucl | 754 | 0.103 | 0.184 | 0.006 | 0.856 | _ | 2 | 0.02 | 0.12 | 0 | 0.86 |
| Gorai.006G057400 | CDKD-1 | cyto | 413 | 0.044 | 0.176 | 0.097 | 0.89 | _ | 2 | 0.01 | 0.05 | 0 | 0.94 |
| Gorai.006G193300 | CDKD-1 | cyto | 410 | 0.033 | 0.17 | 0.088 | 0.918 | _ | 2 | 0.01 | 0.05 | 0 | 0.94 |
| Gorai.006G206400 | CDKE-1 | cyto | 480 | 0.215 | 0.078 | 0.055 | 0.855 | _ | 2 | 0.01 | 0.06 | 0 | 0.93 |
| Gorai.008G205000 | CDKG-2 | chlo | 635 | 0.094 | 0.265 | 0.168 | 0.193 | M | 5 | 0.09 | 0.14 | 0.01 | 0.76 |
| Gorai.008G220700 | CDKF-4 | cyto | 436 | 0.031 | 0.227 | 0.025 | 0.908 | _ | 2 | 0.01 | 0.09 | 0 | 0.89 |
| Gorai.009G026100 | CDKF-1 | cyto | 344 | 0.109 | 0.079 | 0.06 | 0.909 | _ | 1 | 0.01 | 0.06 | 0 | 0.93 |
| Gorai.012G047600 | CDKB1-2 | cyto | 307 | 0.152 | 0.111 | 0.054 | 0.906 | _ | 2 | 0.01 | 0.05 | 0 | 0.94 |
| Gorai.012G174900 | CDKG-2 | nucl | 744 | 0.197 | 0.188 | 0.004 | 0.528 | _ | 4 | 0.02 | 0.14 | 0.01 | 0.83 |
| Gorai.013G013100 | CDKE-1 | cyto | 479 | 0.275 | 0.073 | 0.044 | 0.833 | _ | 3 | 0.01 | 0.06 | 0 | 0.92 |

Len: sequence length; nucl: nucleus; plas: plasma membrane; cyto: cytoplasm; chlo: chloroplast; cTP: chloroplast transit protein; mTP: mitochondrion targeting peptide; C: chloroplast; M: mitochondrion; S: secretory pathway; -: any other location; Loc: location; RC: reliability class, where 1 indicates the strongest prediction (1: diff>0.8; 2:0.8>diff<0.6; 3:0.6>diff>0.4; 4:0.4>diff>0.2 and 5:0.2>diff); diff: the difference the highest and the second highest output score. The lower the RC the higher the accuracy of prediction
