# Supplementary material for: Whole Genome Analysis of Cyclin Dependent Kinase (CDK) Gene Family in Cotton and Functional Evaluation of the Role of CDKF4 Gene in Drought and Salt Stress Tolerance in Plants
Source: Int J Mol Sci. 2018 Sep 5;19(9):2625. doi: 10.3390/ijms19092625 (PMC6164816; doi:10.3390/ijms19092625)
Supplement: Supplementary file 1 [file ijms-19-02625-s001.zip › Supplementary materials/Supplementary Table 3 The details of the 20 motifs identified among the cotton CDKs.docx]

Supplementary Table 3: The details of the 20 motifs identified among the cotton CDKs

| Motif | Best possible match | Motif length (AA) |
| --- | --- | --- |
| 1 | WYRAPELLLGAKQYTSAVDMWSVGCIFAELLTLRPLFPGTSEIDQLGKIF | 50 |
| 2 | GLAYCHDNWVLHRDLKPSNLLV | 22 |
| 3 | AFDLLNKLLTYDPEKRITAAEALNHEYFREEPLPKPKAFLP | 41 |
| 4 | YLVFEYMEHDLYALMKDRKKKFSEVDIKC | 29 |
| 5 | QGVLKJADFGLARIYGSPLKP | 21 |
| 6 | IGEGTYGVVYRARDK | 15 |
| 7 | TALREIKILRELNHPNI | 17 |
| 8 | SVLGTPTEEIWPGFSKLPDYK | 21 |
| 9 | KTGEIVAJKKVKKKK | 15 |
| 10 | ANYSKHQYNLLRKKFPAASFTGSPVLSDA | 29 |
| 11 | MMDBGVQQPVGKKRKFSPIVWDVEDKEVRISSKNRAVVAVT | 41 |
| 12 | AAVSRTPPSVGVRGTLEQQSPRRFSGVLPNAKLTGNFSSLK | 41 |
| 13 | DEQQLDGKEYVPAPNISTSRWASDSDDED | 29 |
| 14 | KSERGELERERLEGNRAQSSERDESVGEARSAVGDDAKKDD | 41 |
| 15 | MLQGCRSVDEFERLN | 15 |
| 16 | TSIEKDRRAIGASDVAEKLANVTIGNRRQNPGQIRAPDVKA | 41 |
| 17 | NGGSLSSSSRNSGGGDGGGGGSDGPKRCEFSGRVVDKEPGPLSSESGSED | 50 |
| 18 | GTGVQRKPEMLNQDLRKNDKPLKNYAKQPRYWPPGRKTPTI | 41 |
| 19 | YPFPKSPGIHLSPQIPSASDD | 21 |
| 20 | TFPAQHAQDRRMRRMLKSPDPLZEQRRKELQQGELGTGGLF | 41 |

Each letter represents a specific amino acid: **A:** Alanine; **C:** Cysteine; **D:** Aspartic acid; **E:** Glutamic acid; **F:**Phenyl alanine; **G:** Glycine**; H:** Histidine; **I:** Isoleucine; **K:** Lysine; **L:**Leucine; **M:** Methionine; **N:** Asparagine; **P:**Proline; **Q:**Glutamine; **R:**Arginine; **S:**Serine; **T:**Threonine; **V:** Valine; **W:**Tryptophan and **Y:** Tyrosine.
