# Supplementary material for: Whole Genome Analysis of Cyclin Dependent Kinase (CDK) Gene Family in Cotton and Functional Evaluation of the Role of CDKF4 Gene in Drought and Salt Stress Tolerance in Plants
Source: Int J Mol Sci. 2018 Sep 5;19(9):2625. doi: 10.3390/ijms19092625 (PMC6164816; doi:10.3390/ijms19092625)
Supplement: Supplementary file 1 [file ijms-19-02625-s001.zip › Supplementary materials/Supplementary Table 4 CDK genes duplication among the three cotton genomes.docx]

Supplementary Table 4: *CDK* genes duplication among the three cotton genomes

| paralogous gene pairs | | Sd | Sn | S | N | ps | pn | dS | dN | dS/dN | ps/pn | Negative/purifying selection |
| --- | --- | --- | --- | --- | --- | --- | --- | --- | --- | --- | --- | --- |
| A | B |  |  |  |  |  |  |  |  |  |  |  |
| Gh-A13G0039 | Gh-A07G00400 | 12.50 | 46.50 | 26.67 | 105.33 | 0.47 | 0.44 | 0.74 | 0.67 | 1.10 | 1.06 | No |
| Cotton_A_10347 | Gorai.001G006600 | 4.50 | 18.50 | 305.33 | 1023.67 | 0.01 | 0.02 | 0.01 | 0.02 | 0.81 | 0.82 | Yes |
| Gorai.001G006600 | Gh-D07G0069 | 1.00 | 4.00 | 302.67 | 1014.33 | 0.00 | 0.00 | 0.00 | 0.00 | 0.84 | 0.84 | Yes |
| Gorai.001G006601 | Gh-D05G0242 | 28.17 | 86.83 | 127.83 | 463.17 | 0.22 | 0.19 | 0.26 | 0.22 | 1.21 | 1.18 | No |
| Gorai.001G006602 | Cotton_A_11170 | 51.83 | 190.17 | 260.00 | 892.00 | 0.20 | 0.21 | 0.23 | 0.25 | 0.92 | 0.94 | Yes |
| Gorai.001G006603 | Gh-A05G0178 | 38.67 | 133.33 | 204.67 | 689.33 | 0.19 | 0.19 | 0.22 | 0.22 | 0.97 | 0.98 | Yes |
| Gorai.001G006604 | Gh-D12G2017 | 83.67 | 342.33 | 171.00 | 606.00 | 0.49 | 0.56 | 0.79 | 1.05 | 0.76 | 0.87 | Yes |
| Gorai.001G006605 | Cotton_A_07964 | 80.67 | 338.33 | 168.50 | 602.50 | 0.48 | 0.56 | 0.76 | 1.04 | 0.74 | 0.85 | Yes |
| Gorai.001G006606 | Gorai.008G220700 | 82.17 | 340.83 | 170.67 | 606.33 | 0.48 | 0.56 | 0.77 | 1.04 | 0.74 | 0.86 | Yes |
| Gorai.001G006607 | Gh-A08G1357 | 97.17 | 390.83 | 198.00 | 693.00 | 0.49 | 0.56 | 0.80 | 1.05 | 0.76 | 0.87 | Yes |
| Gorai.001G006608 | Gh-D08G1653 | 81.83 | 344.17 | 170.50 | 606.50 | 0.48 | 0.57 | 0.77 | 1.06 | 0.72 | 0.85 | Yes |
| Gorai.001G006609 | Cotton_A_14275 | 82.17 | 342.83 | 169.50 | 607.50 | 0.48 | 0.56 | 0.78 | 1.05 | 0.74 | 0.86 | Yes |
| Gorai.001G006610 | Gorai.004G178800 | 81.83 | 344.17 | 170.17 | 606.83 | 0.48 | 0.57 | 0.77 | 1.06 | 0.73 | 0.85 | Yes |
| Gorai.001G006611 | Gorai.003G187100 | 77.00 | 328.00 | 159.83 | 584.17 | 0.48 | 0.56 | 0.77 | 1.04 | 0.74 | 0.86 | Yes |
| Gorai.001G006612 | Gh-D03G1838 | 78.33 | 338.67 | 165.83 | 608.17 | 0.47 | 0.56 | 0.75 | 1.02 | 0.73 | 0.85 | Yes |
| Gorai.001G006613 | Gh-A03G1965 | 79.00 | 343.00 | 167.67 | 618.33 | 0.47 | 0.55 | 0.74 | 1.01 | 0.74 | 0.85 | Yes |
| Gorai.001G006614 | Cotton_A_25379 | 78.83 | 341.17 | 166.17 | 607.83 | 0.47 | 0.56 | 0.75 | 1.03 | 0.73 | 0.85 | Yes |
| Gorai.001G006615 | Gh-A12G1847 | 82.00 | 340.00 | 170.33 | 603.67 | 0.48 | 0.56 | 0.77 | 1.04 | 0.74 | 0.85 | Yes |
| Gorai.001G006616 | Gh-D02G1543 | 91.83 | 342.17 | 188.83 | 696.17 | 0.49 | 0.49 | 0.78 | 0.80 | 0.98 | 0.99 | Yes |
| Gorai.001G006617 | Gorai.005G170300 | 92.83 | 342.17 | 188.83 | 696.17 | 0.49 | 0.49 | 0.80 | 0.80 | 1.00 | 1.00 | No |
| Gorai.001G006618 | Gh-A03G1115 | 91.50 | 342.50 | 188.17 | 696.83 | 0.49 | 0.49 | 0.78 | 0.80 | 0.98 | 0.99 | Yes |
| Gorai.001G006619 | Gh-A13G0098 | 100.17 | 348.83 | 187.67 | 643.33 | 0.53 | 0.54 | 0.93 | 0.96 | 0.97 | 0.98 | Yes |
| Gorai.001G006620 | Gh-D13G0113 | 99.50 | 355.50 | 189.83 | 647.17 | 0.52 | 0.55 | 0.90 | 0.99 | 0.91 | 0.95 | Yes |
| Cotton_A_07964. | Cotton_A_01035 | 101.33 | 339.67 | 196.17 | 694.83 | 0.52 | 0.49 | 0.88 | 0.79 | 1.11 | 1.06 | No |
| Cotton_A_10347. | Cotton_A_01035 | 98.50 | 352.50 | 187.50 | 643.50 | 0.53 | 0.55 | 0.90 | 0.98 | 0.92 | 0.96 | Yes |
| Cotton_A_11170. | Cotton_A_01035 | 82.67 | 299.33 | 159.33 | 557.67 | 0.52 | 0.54 | 0.88 | 0.94 | 0.94 | 0.97 | Yes |
| Cotton_A_14275. | Cotton_A_01035 | 102.00 | 350.00 | 199.83 | 703.17 | 0.51 | 0.50 | 0.86 | 0.82 | 1.05 | 1.03 | No |
| Cotton_A_25379. | Cotton_A_01035 | 96.17 | 354.83 | 196.33 | 706.67 | 0.49 | 0.50 | 0.79 | 0.83 | 0.96 | 0.98 | Yes |
| Gh-A03G1115 | Cotton_A_01035 | 84.33 | 311.67 | 187.00 | 716.00 | 0.45 | 0.44 | 0.69 | 0.65 | 1.06 | 1.04 | No |
| Gh-A03G1965 | Cotton_A_01035 | 100.00 | 364.00 | 200.83 | 729.17 | 0.50 | 0.50 | 0.82 | 0.82 | 1.00 | 1.00 | Yes |
| Gh-A05G0178 | Cotton_A_01035 | 66.33 | 195.67 | 110.67 | 372.33 | 0.60 | 0.53 | 1.20 | 0.90 | 1.33 | 1.14 | No |
| Gh-A08G1357 | Cotton_A_01035 | 106.17 | 365.83 | 209.33 | 732.67 | 0.51 | 0.50 | 0.85 | 0.82 | 1.03 | 1.02 | No |
| Gh-A12G1847 | Cotton_A_01035 | 99.33 | 339.67 | 198.00 | 693.00 | 0.50 | 0.49 | 0.83 | 0.79 | 1.04 | 1.02 | No |
| Gh-A13G0098 | Cotton_A_01035 | 2.00 | 1.00 | 278.17 | 990.83 | 0.01 | 0.00 | 0.01 | 0.00 | 7.15 | 7.12 | No |
| Gh-D02G1543 | Cotton_A_01035 | 85.50 | 308.50 | 187.33 | 715.67 | 0.46 | 0.43 | 0.70 | 0.64 | 1.10 | 1.06 | No |
| Gh-D03G1838 | Cotton_A_01035 | 97.00 | 350.00 | 196.00 | 707.00 | 0.49 | 0.50 | 0.81 | 0.81 | 1.00 | 1.00 | Yes |
| Gh-D05G0242 | Cotton_A_01035 | 36.50 | 167.50 | 87.50 | 308.50 | 0.42 | 0.54 | 0.61 | 0.97 | 0.63 | 0.77 | Yes |
| Gh-D07G0069 | Cotton_A_01035 | 99.17 | 347.83 | 187.00 | 641.00 | 0.53 | 0.54 | 0.92 | 0.96 | 0.96 | 0.98 | Yes |
| Gh-D08G1653 | Cotton_A_01035 | 105.33 | 349.67 | 201.00 | 705.00 | 0.52 | 0.50 | 0.90 | 0.81 | 1.11 | 1.06 | No |
| Gh-D12G2017 | Cotton_A_01035 | 97.33 | 336.67 | 198.17 | 692.83 | 0.49 | 0.49 | 0.80 | 0.78 | 1.02 | 1.01 | No |
| Gh-D13G0113 | Cotton_A_01035 | 2.00 | 15.00 | 279.83 | 992.17 | 0.01 | 0.02 | 0.01 | 0.02 | 0.47 | 0.47 | Yes |
| Gorai.001G006621 | Cotton_A_01035 | 100.17 | 348.83 | 187.67 | 643.33 | 0.53 | 0.54 | 0.93 | 0.96 | 0.97 | 0.98 | Yes |
| Gorai.003G187100 | Cotton_A_01035 | 91.17 | 336.83 | 187.33 | 673.67 | 0.49 | 0.50 | 0.79 | 0.82 | 0.95 | 0.97 | Yes |
| Gorai.004G178800 | Cotton_A_01035 | 105.50 | 348.50 | 200.67 | 705.33 | 0.53 | 0.49 | 0.91 | 0.81 | 1.12 | 1.06 | No |
| Gorai.005G170300 | Cotton_A_01035 | 85.50 | 309.50 | 187.00 | 716.00 | 0.46 | 0.43 | 0.71 | 0.64 | 1.10 | 1.06 | No |
| Gorai.008G220700 | Cotton_A_01035 | 96.83 | 343.17 | 202.00 | 704.00 | 0.48 | 0.49 | 0.76 | 0.79 | 0.97 | 0.98 | Yes |
| Gorai.009G026100 | Cotton_A_01035 | 59.83 | 215.17 | 122.83 | 417.17 | 0.49 | 0.52 | 0.79 | 0.87 | 0.90 | 0.94 | Yes |
| Gorai.001G006622 | Gh-A09G1688 | 94.67 | 349.33 | 187.67 | 649.33 | 0.50 | 0.54 | 0.84 | 0.95 | 0.88 | 0.94 | Yes |
| Gorai.001G006623 | Gorai.006G206400 | 96.67 | 350.33 | 187.67 | 649.33 | 0.52 | 0.54 | 0.87 | 0.95 | 0.91 | 0.95 | Yes |
| Gorai.001G006624 | Cotton_A_08146 | 95.17 | 350.83 | 188.17 | 648.83 | 0.51 | 0.54 | 0.84 | 0.96 | 0.88 | 0.94 | Yes |
| Gorai.001G006625 | Gh-D09G1794 | 96.17 | 347.83 | 187.67 | 649.33 | 0.51 | 0.54 | 0.86 | 0.94 | 0.92 | 0.96 | Yes |
| Gorai.001G006626 | Gorai.013G013100 | 99.33 | 357.67 | 189.33 | 647.67 | 0.52 | 0.55 | 0.90 | 1.00 | 0.90 | 0.95 | Yes |
| Gorai.001G006627 | Gorai.006G193300 | 86.17 | 313.83 | 164.00 | 586.00 | 0.53 | 0.54 | 0.90 | 0.94 | 0.96 | 0.98 | Yes |
| Gorai.001G006628 | Gh-A09G1581 | 86.17 | 315.83 | 164.33 | 585.67 | 0.52 | 0.54 | 0.90 | 0.95 | 0.95 | 0.97 | Yes |
| Gorai.001G006629 | Cotton_A_13038 | 86.83 | 313.17 | 164.50 | 585.50 | 0.53 | 0.53 | 0.91 | 0.94 | 0.97 | 0.99 | Yes |
| Gorai.001G006630 | Gh-D09G1668 | 85.83 | 312.17 | 163.17 | 586.83 | 0.53 | 0.53 | 0.91 | 0.93 | 0.98 | 0.99 | Yes |
| Gorai.001G006631 | Gh-D09G0505 | 86.17 | 318.83 | 163.33 | 592.67 | 0.53 | 0.54 | 0.91 | 0.95 | 0.96 | 0.98 | Yes |
| Gorai.001G006632 | Gorai.006G057400 | 86.17 | 318.83 | 163.33 | 592.67 | 0.53 | 0.54 | 0.91 | 0.95 | 0.96 | 0.98 | Yes |
| Gorai.001G006633 | Cotton_A_27620 | 86.67 | 316.33 | 162.00 | 588.00 | 0.54 | 0.54 | 0.94 | 0.95 | 0.99 | 0.99 | Yes |
| Gorai.001G006634 | Gh-A09G0498 | 87.33 | 318.67 | 162.83 | 593.17 | 0.54 | 0.54 | 0.94 | 0.94 | 1.00 | 1.00 | Yes |
| Gorai.001G006635 | Gh-A08G1333 | 98.83 | 335.17 | 180.50 | 647.50 | 0.55 | 0.52 | 0.98 | 0.88 | 1.12 | 1.06 | No |
| Gorai.001G006636 | Gorai.004G175700 | 99.67 | 334.33 | 181.17 | 646.83 | 0.55 | 0.52 | 0.99 | 0.88 | 1.13 | 1.06 | No |
| Gorai.001G006637 | Cotton_A_13019 | 99.83 | 335.17 | 181.50 | 652.50 | 0.55 | 0.51 | 0.99 | 0.87 | 1.14 | 1.07 | No |
| Gorai.001G006638 | Gh-D08G1628 | 99.67 | 333.33 | 182.83 | 651.17 | 0.55 | 0.51 | 0.97 | 0.86 | 1.13 | 1.06 | No |
| Gorai.001G006639 | Cotton_A_33907 | 84.00 | 322.00 | 177.67 | 662.33 | 0.47 | 0.49 | 0.75 | 0.78 | 0.95 | 0.97 | Yes |
| Gorai.001G006640 | Gh-A04G1202 | 82.83 | 346.17 | 187.00 | 692.00 | 0.44 | 0.50 | 0.67 | 0.82 | 0.81 | 0.89 | Yes |
| Gorai.001G006641 | Cotton_A_14138 | 83.33 | 350.67 | 189.17 | 695.83 | 0.44 | 0.50 | 0.66 | 0.84 | 0.79 | 0.87 | Yes |
| Gorai.001G006642 | Gh-D07G0534 | 84.83 | 340.17 | 169.83 | 643.17 | 0.50 | 0.53 | 0.82 | 0.92 | 0.90 | 0.94 | Yes |
| Gorai.001G006643 | Gorai.001G060800 | 83.67 | 342.33 | 170.67 | 642.33 | 0.49 | 0.53 | 0.80 | 0.93 | 0.86 | 0.92 | Yes |
| Gorai.001G006644 | Gh-A07G0469 | 83.67 | 341.33 | 171.00 | 642.00 | 0.49 | 0.53 | 0.79 | 0.93 | 0.86 | 0.92 | Yes |
| Gorai.001G006645 | Cotton_A_19907 | 82.50 | 341.50 | 169.83 | 643.17 | 0.49 | 0.53 | 0.78 | 0.92 | 0.85 | 0.91 | Yes |
| Cotton_A_01035. | Cotton_A_08058 | 69.83 | 291.17 | 167.50 | 645.50 | 0.42 | 0.45 | 0.61 | 0.69 | 0.88 | 0.92 | Yes |
| Cotton_A_08146 | Cotton_A_08058 | 73.17 | 289.83 | 166.33 | 646.67 | 0.44 | 0.45 | 0.66 | 0.68 | 0.97 | 0.98 | Yes |
| Cotton_A_13019 | Cotton_A_08058 | 64.83 | 291.17 | 168.00 | 645.00 | 0.39 | 0.45 | 0.54 | 0.69 | 0.78 | 0.85 | Yes |
| Cotton_A_13038 | Cotton_A_08058 | 69.17 | 300.83 | 158.17 | 606.83 | 0.44 | 0.50 | 0.66 | 0.81 | 0.81 | 0.88 | Yes |
| Cotton_A_14138 | Cotton_A_08058 | 119.17 | 413.83 | 311.67 | 1266.33 | 0.38 | 0.33 | 0.53 | 0.43 | 1.25 | 1.17 | No |
| Cotton_A_19907 | Cotton_A_08058 | 46.33 | 189.67 | 273.33 | 1124.67 | 0.17 | 0.17 | 0.19 | 0.19 | 1.01 | 1.01 | No |
| Cotton_A_27620 | Cotton_A_08058 | 69.50 | 306.50 | 156.50 | 608.50 | 0.44 | 0.50 | 0.67 | 0.84 | 0.81 | 0.88 | Yes |
| Cotton_A_33907 | Cotton_A_08058 | 128.67 | 441.33 | 299.67 | 1221.33 | 0.43 | 0.36 | 0.64 | 0.49 | 1.29 | 1.19 | No |
| Gh-A04G1202 | Cotton_A_08058 | 118.83 | 417.17 | 312.00 | 1266.00 | 0.38 | 0.33 | 0.53 | 0.43 | 1.23 | 1.16 | No |
| Gh-A07G0469 | Cotton_A_08058 | 57.67 | 216.33 | 307.33 | 1246.67 | 0.19 | 0.17 | 0.22 | 0.20 | 1.09 | 1.08 | No |
| Gh-A08G1333 | Cotton_A_08058 | 66.33 | 290.67 | 168.67 | 644.33 | 0.39 | 0.45 | 0.56 | 0.69 | 0.81 | 0.87 | Yes |
| Gh-A09G0498 | Cotton_A_08058 | 70.83 | 309.17 | 157.50 | 616.50 | 0.45 | 0.50 | 0.69 | 0.83 | 0.83 | 0.90 | Yes |
| Gh-A09G1581 | Cotton_A_08058 | 70.83 | 299.17 | 158.00 | 607.00 | 0.45 | 0.49 | 0.68 | 0.80 | 0.85 | 0.91 | Yes |
| Gh-A09G1688 | Cotton_A_08058 | 71.67 | 288.33 | 165.33 | 647.67 | 0.43 | 0.45 | 0.65 | 0.68 | 0.96 | 0.97 | Yes |
| Gh-D07G0534 | Cotton_A_08058 | 57.17 | 208.83 | 305.33 | 1248.67 | 0.19 | 0.17 | 0.22 | 0.19 | 1.14 | 1.12 | No |
| Gh-D08G1628 | Cotton_A_08058 | 68.00 | 292.00 | 169.67 | 643.33 | 0.40 | 0.45 | 0.57 | 0.70 | 0.82 | 0.88 | Yes |
| Gh-D09G0505 | Cotton_A_08058 | 70.67 | 305.33 | 158.00 | 616.00 | 0.45 | 0.50 | 0.68 | 0.81 | 0.84 | 0.90 | Yes |
| Gh-D09G1668 | Cotton_A_08058 | 71.67 | 297.33 | 156.67 | 608.33 | 0.46 | 0.49 | 0.71 | 0.79 | 0.89 | 0.94 | Yes |
| Gh-D09G1794 | Cotton_A_08058 | 71.17 | 287.83 | 165.67 | 647.33 | 0.43 | 0.44 | 0.64 | 0.67 | 0.95 | 0.97 | Yes |
| Gorai.001G006646 | Cotton_A_08058 | 90.50 | 340.50 | 173.83 | 642.17 | 0.52 | 0.53 | 0.89 | 0.92 | 0.97 | 0.98 | Yes |
| Gorai.001G060800 | Cotton_A_08058 | 54.17 | 205.83 | 306.00 | 1248.00 | 0.18 | 0.16 | 0.20 | 0.19 | 1.08 | 1.07 | No |
| Gorai.004G175700 | Cotton_A_08058 | 67.00 | 292.00 | 169.00 | 644.00 | 0.40 | 0.45 | 0.56 | 0.70 | 0.81 | 0.87 | Yes |
| Gorai.006G057400 | Cotton_A_08058 | 70.67 | 307.33 | 158.17 | 615.83 | 0.45 | 0.50 | 0.68 | 0.82 | 0.83 | 0.90 | Yes |
| Gorai.006G193300 | Cotton_A_08058 | 72.67 | 298.33 | 158.00 | 607.00 | 0.46 | 0.49 | 0.71 | 0.80 | 0.89 | 0.94 | Yes |
| Gorai.006G206400 | Cotton_A_08058 | 72.67 | 289.33 | 166.17 | 646.83 | 0.44 | 0.45 | 0.66 | 0.68 | 0.96 | 0.98 | Yes |
| Gorai.013G013100 | Cotton_A_08058 | 72.33 | 297.67 | 168.17 | 644.83 | 0.43 | 0.46 | 0.64 | 0.72 | 0.89 | 0.93 | Yes |
| Gorai.001G006647 | Gh-A12G1705 | 91.33 | 340.67 | 174.00 | 642.00 | 0.52 | 0.53 | 0.90 | 0.92 | 0.98 | 0.99 | Yes |
| Gh-A12G1705 | Gorai.008G205000 | 4.67 | 39.33 | 324.00 | 1329.00 | 0.01 | 0.03 | 0.01 | 0.03 | 0.48 | 0.49 | Yes |
| Gorai.001G006648 | Gorai.008G205000 | 88.67 | 340.33 | 173.33 | 642.67 | 0.51 | 0.53 | 0.86 | 0.92 | 0.94 | 0.97 | Yes |
| Gorai.001G006649 | Gh-D12G1867 | 77.17 | 304.83 | 155.50 | 585.50 | 0.50 | 0.52 | 0.81 | 0.89 | 0.91 | 0.95 | Yes |
| Gorai.001G006650 | Gorai.012G174900 | 82.17 | 346.83 | 187.83 | 688.17 | 0.44 | 0.50 | 0.66 | 0.84 | 0.79 | 0.87 | Yes |
| Gorai.001G006651 | Gh-D04G1812 | 82.17 | 346.83 | 187.83 | 688.17 | 0.44 | 0.50 | 0.66 | 0.84 | 0.79 | 0.87 | Yes |
| Gorai.001G006652 | Gh-A09G0392 | 21.17 | 124.83 | 63.00 | 228.00 | 0.34 | 0.55 | 0.45 | 0.98 | 0.45 | 0.61 | Yes |
| Cotton_A_08058. | Cotton_A_27238 | 65.67 | 272.33 | 165.83 | 596.17 | 0.40 | 0.46 | 0.56 | 0.70 | 0.80 | 0.87 | Yes |
| Gh_A04G1202 | Cotton_A_27238 | 67.00 | 264.00 | 168.50 | 593.50 | 0.40 | 0.44 | 0.57 | 0.67 | 0.84 | 0.89 | Yes |
| Gh_A12G1705 | Cotton_A_27238 | 66.50 | 271.50 | 165.83 | 596.17 | 0.40 | 0.46 | 0.57 | 0.70 | 0.82 | 0.88 | Yes |
| Gh_D02G1543 | Cotton_A_27238 | 70.67 | 266.33 | 168.67 | 596.33 | 0.42 | 0.45 | 0.61 | 0.68 | 0.90 | 0.94 | Yes |
| Gh_D05G0242 | Cotton_A_27238 | 52.83 | 187.17 | 94.83 | 313.17 | 0.56 | 0.60 | 1.02 | 1.20 | 0.85 | 0.93 | Yes |
| Gh_D07G0534 | Cotton_A_27238 | 71.17 | 281.83 | 165.00 | 597.00 | 0.43 | 0.47 | 0.64 | 0.74 | 0.86 | 0.91 | Yes |
| Gh-A09G0392 | Cotton_A_27238 | 1.00 | 1.00 | 74.67 | 258.33 | 0.01 | 0.00 | 0.01 | 0.00 | 3.48 | 3.46 | No |
| Gh-D04G1812 | Cotton_A_27238 | 66.00 | 265.00 | 170.17 | 597.83 | 0.39 | 0.44 | 0.55 | 0.67 | 0.81 | 0.88 | Yes |
| Gh-D12G1867 | Cotton_A_27238 | 54.17 | 250.83 | 147.50 | 536.50 | 0.37 | 0.47 | 0.50 | 0.73 | 0.69 | 0.79 | Yes |
| Gorai.001G006653 | Cotton_A_27238 | 73.83 | 304.17 | 153.17 | 524.83 | 0.48 | 0.58 | 0.77 | 1.11 | 0.69 | 0.83 | Yes |
| Gorai.008G205000 | Cotton_A_27238 | 66.17 | 270.83 | 165.67 | 596.33 | 0.40 | 0.45 | 0.57 | 0.70 | 0.82 | 0.88 | Yes |
| Gorai.012G174900 | Cotton_A_27238 | 66.00 | 265.00 | 170.17 | 597.83 | 0.39 | 0.44 | 0.55 | 0.67 | 0.81 | 0.88 | Yes |
| Cotton_A_27238. | Gh-D04G0378 | 1.00 | 4.00 | 202.17 | 637.83 | 0.00 | 0.01 | 0.01 | 0.01 | 0.79 | 0.79 | Yes |
| Gorai.001G006654 | Gh-D04G0378 | 73.50 | 305.50 | 153.33 | 524.67 | 0.48 | 0.58 | 0.76 | 1.12 | 0.68 | 0.82 | Yes |
| Gh-D04G0378 | Gorai.012G047600 | 1.00 | 1.00 | 202.50 | 637.50 | 0.00 | 0.00 | 0.01 | 0.00 | 3.16 | 3.15 | No |
| Gorai.001G006655 | Gorai.012G047600 | 73.33 | 304.67 | 153.50 | 524.50 | 0.48 | 0.58 | 0.76 | 1.12 | 0.68 | 0.82 | Yes |

Sd: standard deviation; s: number of synonymous sites; n: number of nonsynonymous sites; S: number of synonymous substitutions; N: number of nonsynonymous substitutions; dS: synonymous substitution rate; dN: nonsynonymous substitution; dS/dN: selective strength of sequence; ps: probability of rejecting the null hypothesis
