# Supplementary material for: Whole Genome Analysis of Cyclin Dependent Kinase (CDK) Gene Family in Cotton and Functional Evaluation of the Role of CDKF4 Gene in Drought and Salt Stress Tolerance in Plants
Source: Int J Mol Sci. 2018 Sep 5;19(9):2625. doi: 10.3390/ijms19092625 (PMC6164816; doi:10.3390/ijms19092625)
Supplement: Supplementary file 1 [file ijms-19-02625-s001.zip › Supplementary materials/Supplementary Table 5 List of CDK genes specific primers used in the validation of expression profile of the cotton genomes CDK gen.docx]

Supplementary Table 8: List of *CDK* genes specific primers used in the validation of expression profile of the cotton genomes *CDK* genes under salt and drought stress

| *G. raimondii* | FORWARD | REVERSE |  |
| --- | --- | --- | --- |
| Gorai.001G006600 | ACCTCATCGTCGCCCTCA | CCCGCCAAAAATACTCGT | |
| Gorai.001G060800 | GGGTTCAATTTGGGTCCTA | TTCCTCTTTTTCTGTGCGG | |
| Gorai.003G187100 | TTCACCTTACATCCTCTTTT | TCTTCAGTTGGAGTGCCTAC | |
| Gorai.004G175700 | CTGACCCATTACCGTGCG | ATGCTGTAGTTTCTGCCG | |
| Gorai.004G178800 | CACCAGGAAGAAGGACAAC | CTCCGCCACCTCTGAAGCC | |
| Gorai.005G170300 | ACCACAAGTCACCTAACA | CAGTGCAGAGTATCCAAC | |
| Gorai.006G057400 | GCACCTCCACCTACCAATC | ACCTTCAAACCTCTCACGA | |
| Gorai.006G193300 | TTTTCATCTGCTCCTCTAC | ACGATCTGGCATTACTCTC | |
| Gorai.006G206400 | GAAATAACACCTCAGGCAGA | AGCAAGAAACACAAGACCAT | |
| Gorai.008G205000 | CCTCTTAAGCCTTACACA | TACATCCCACAGACCACA | |
| Gorai.008G220700 | GCCACATCCATCAACAAGG | CAGTCCACAGAACCCCAGC | |
| Gorai.009G026100 | ATGAGGCTGAAGATGGGGA | CATAGAGTAGTTCGGGGGC | |
| Gorai.012G047600 | TGCTTGGCTCTACTCACT | TTGCCTTCTAACCATTTC | |
| Gorai.012G174900 | GCAATGGATAATGGGGCT | ACTGCTGGTGGAGGAGGA | |
| Gorai.013G013100 | AGGCTGGAGTGAGTGGTGG | TTTTTGCTGCGGTGGGTAT | |
| *G. arboreum* | **FORWARD** | **REVERSE** |  |
| Cotton_A_01035 | AGGCTGGAGTGAGTGGTGG | TTTTTGCTGCGGTGGGTAT | |
| Cotton_A_07964 | GCCACATCCATCAACAAGG | CAGTCCACAGAACCCCAGC | |
| Cotton_A_08058 | ATTGTGGCACTGAAGAAG | AAGGGTGATGAAAAGACG | |
| Cotton_A_10347 | ACCTCATCGTCGCCCTCA | CCCGCCAAAAATACTCGT | |
| Cotton_A_11170 | GAAGGGGATGACAGAAAA | AAAAGGGGCTTAAGGGTT | |
| Cotton_A_13019 | CTGACCCATTACCATGCGAT | GGATGCTGTAGTTTCTGCCG | |
| Cotton_A_13038 | TTTTCATCTGCTCCTCTA | ATCTGGCATTACTCTCCT | |
| Cotton_A_13039 | GCCTACCCCTAAATCTCGT | GGTCATCAATCTTCTCCAC | |
| Cotton_A_14138 | GCAATGGATAATGGGGCT | ACTGCTGGTGGAGGAGGA | |
| Cotton_A_14275 | GCCCTTCAACATCCATTC | GTTCCTCTAACCCCAGCA | |
| Cotton_A_19907 | CTCAAAAGCCATATACAA | ATACAGCCTACAGACCAC | |
| Cotton_A_25379 | TTCACCTTACATCCTCTTTT | TTTTCAGTTGGAGTGCCTAC | |
| *G. hirsutum* | **FORWARD** | **REVERSE** |  |
| Gh_A03G1115 | ACCACAAGTCACCTAACA | CAGTGCAGAGTATCCAAC | |
| Gh_A03G1965 | GAACACGAACTCGGAACACC | CTTGAGAATCCATACACCAT | |
| Gh_A04G1202 | GCAATGGATAATGGGGCT | ACTGCTGGTGGAGGAGGA | |
| Gh_A05G0178 | AAGGGGATGACAGAAAAG | TTGGAAGAGGAAGAGGTT | |
| Gh_A07G0040 | TCAGAGCCCCGGAACTAC | GAAACCCCAGGAAAAAGC | |
| Gh_A07G0469 | ATGGGTTCAATTTGGGTCC | TTCCTCTTTTTCTGTGCGG | |
| Gh_A08G1333 | CTGACCCATTACCATGCGAT | GGATGCTGTAGTTTCTGCCG | |
| Gh_A08G1357 | TGGAAGTCGGAGGCAGTATG | GACGGTATCTGTTGGGTTGG | |
| Gh_A09G0392 | CACCCCAACTGAGAAGCA | CAGGAGAAAGGGAAGGAA | |
| Gh_A09G0498 | TCTGGTGTGGATGTTTGG | TGAGAAGATGTTGGGGTC | |
| Gh_A09G1581 | TTTTCATCTGCTCCTCTA | ATCTGGCATTACTCTCCT | |
| Gh_A09G1688 | GAAATAACAACTCAGGCAG | TAGCAAGAAACACAAGACC | |
| Gh_A12G1705 | AGTTCGATCTGGGTCCTA | ACTTCCTCTTTTTTTCCG | |
| Gh_A12G1847 | GCCACATCCATCAACAAGG | CAGTCCACAGAACCCCAGC | |
| Gh_A13G0098 | AGGCTGGAGTGAGTGGTGG | TTTTGCTGCGGTGGGTATC | |
| Gh_D02G1543 | TCACAACAGATTACGGAA | TGGACTTGGAACGAGCAC | |
| Gh_D03G1838 | CACCTTACATCCTCTTTT | TTCAGTTGGAGTGCCTAC | |
| Gh_D04G0378 | TGCTTGGCTCTACTCACTAC | TTGCCTTCTAACCATTTCGG | |
| Gh_D04G1812 | GCAATGGATAATGGGGCT | ACTGCTGGTGGAGGAGGA | |
| Gh_D05G0242 | GCCCCTGAACTACTCTATGG | AGCTCTGAAAAAATGCAACC | |
| Gh_D07G0069 | ACCTCATCGTCGCCCTCA | CCCGCCAAAAATACTCGT | |
| Gh_D07G0534 | GGGTTCAATTTGGGTCCT | TCCTCTTTTTCTGTGCGG | |
| Gh_D08G1628 | CTGACCCATTACCGTGCG | ATGCTGTAGTTTCTGCCG | |
| Gh_D08G1653 | CACCAGGAAGAAGGACAAC | CTCCGCCACCTCTGAAGCC | |
| Gh_D09G0505 | CTGCACCTCCACCTACCG | CCTTCAAACCTCTCACGA | |
| Gh_D09G1668 | TTTTCATCTGCTCCTCTA | ATCTGGCATTACTCTCCT | |
| Gh_D09G1794 | AATAACACCTCAGGCAGA | AGCAAGAAACACAAGACC | |
| Gh_D12G1867 | AGTTCGATCTGGGTCCTA | ACTTCCTCTTTTTTTCCG | |
| Gh_D12G2017 | GCCACATCCATCGACAAGG | CAGTCCACAGAACCCCAGC | |
| Gh_D13G0113 | AATAACACCTCAGGCAGA | CAAGAAACACGAGACCAT | |
