# Supplementary material for: Whole Genome Analysis of Cyclin Dependent Kinase (CDK) Gene Family in Cotton and Functional Evaluation of the Role of CDKF4 Gene in Drought and Salt Stress Tolerance in Plants
Source: Int J Mol Sci. 2018 Sep 5;19(9):2625. doi: 10.3390/ijms19092625 (PMC6164816; doi:10.3390/ijms19092625)
Supplement: Supplementary file 1 [file ijms-19-02625-s001.zip › Supplementary materials/Supplementary Table 6 Primer details for the abiotic stress responsive gens for RT-qPCR analysis for the response of transgenic an.docx]

**Supplementary Table 9.** Primer details for the abiotic stress responsive gens for RT-qPCR analysis for the response of transgenic and wild type lines under salt and drought stress conditions

| GENE | FORWARD SEQUENCE | REVERSE SEQUENCE |
| --- | --- | --- |
| ABF4 | AACAACTTAGGAGGTGGTGGTCAT | TGTAGCAGCTGGCGCAGAAGTCAT |
| CBL1 | GAAATGAAACTGGCTGATGAAACCATAGAG | CTCGTGGCAATCTACTCGGTCTTAAACC |
| RD29A | TGAAAGGAGGAGGAGGAATGGTTGG | ACAAAACACACATAAACATCCAAAGT |
